# Resonance of the tympanoperiotic complex of fin whales with implications for their low frequency hearing

**DOI:** 10.1371/journal.pone.0288119

**Published:** 2023-10-11

**Authors:** Margaret Morris, Petr Krysl, John Hildebrand, Ted Cranford

**Affiliations:** 1 Scripps Institution of Oceanography, University of California, San Diego, California, United States of Ameirca; 2 Department of Structural Engineering, University of California, San Diego, California, United States of Ameirca; 3 Department of Biology, San Diego State University, San Diego, California, United States of Ameirca; GSVM Medical College, INDIA

## Abstract

The tympanoperiotic complex (TPC) bones of the fin whale skull were studied using experimental measurements and simulation modeling to provide insight into the low frequency hearing of these animals. The study focused on measuring the sounds emitted by the left and right TPC bones when the bones were tapped at designated locations. Radiated sound was recorded by eight microphones arranged around the tympanic bulla. A finite element model was also created to simulate the natural mode vibrations of the TPC and ossicular chain, using a 3D mesh generated from a CT scan. The simulations produced mode shapes and frequencies for various Young’s modulus and density values. The recorded sound amplitudes were compared with the normal component of the simulated displacement and it was found that the modes identified in the experiment most closely resembled those found with Young’s modulus for stiff and flexible bone set to 25 and 5 GPa, respectively. The first twelve modes of vibration of the TPC had resonance frequencies between 100Hz and 6kHz. Many vibrational modes focused energy at the sigmoidal process, and therefore the ossicular chain. The resonance frequencies of the left and right TPC were offset, suggesting a mechanism for the animals to have improved hearing at a range of frequencies as well as a mechanism for directionality in their perception of sounds.

## Introduction

Knowledge of mysticete low frequency hearing sensitivity is needed to assess the potential impact of anthropogenic noise on their anatomy and behavior [1]. This is increasingly important due to the presence of noise from shipping traffic, seismic exploration, and offshore development in whale habitat [2]. Particularly helpful would be knowing the frequency sensitivity of hearing for a variety of different whale species.

Studies of mysticeti hearing are sparse, particularly owing to the difficulty of conducting behavioral or electrophysiological studies. An alternative approach is to numerically model whale hearing capabilities, allowing simulated sound exposure without impacting live animals [3]. A modelling approach also enables studies of the effect of exposure to specific frequencies without the extra noise and extraneous effects that may be present in experiments with live animals.

The tympanoperiotic complex (TPC) plays a crucial role in whale hearing, serving as a functional unit composed of three major bony structures attached to the skull: the periotic, the tympanic bulla (including pedicles), and the ossicular chain [4]. In a pioneering study, Quiralte and Dell [5] used elastic solid vibrations theory to explore whale hearing mechanisms, and focused on a specific component of the TPC, the tympanic bulla detached from the pedicles and the periotic bone. Their finite element analysis revealed low-frequency (LF) mode shapes that displayed the greatest response near the malleus. As a result they concluded that the tympanic bone collects sound vibrations over its surface and concentrates energy at the malleus. However, the natural vibrations of the TPC in a living whale may be influenced by the attachments of the tympanic bulla to the skull through the pedicles and periotic bone. Our research examines the entire TPC and lays the foundation for more comprehensive models.

Our previous work [3] generated synthetic audiograms for a fin whale by applying finite element modeling tools to X-ray computed tomography (CT) scans. The simulations revealed two mechanisms that could excite the bony ear complexes: (1) the skull-vibration enabled bone conduction mechanism, and (2) a pressure mechanism transmitted through soft tissues. Bone conduction was determined to be the more effective mechanism. The mass density of the bony ear complexes and their firmly embedded attachments to the skull were found to be universal across the Mysticeti. Hence, the work hypothesized that sound reception mechanisms were similar in all baleen whales. The interactions between incident sound waves and the skull were thought to induce motion of the bony ear complex relative to the skull, subsequently transmitted through the ossicular chain, resulting in best hearing sensitivity for low-frequency sounds.

In this work, we combine direct measurements of the vibrations of the fin whale bulla with a vibroacoustic finite element model that has been previously used to successfully simulate sound production and sound beam formation produced by odontocetes [6]. We include in the model the entire TPC, with the base of the periotic bone fixed in place to represent rigid attachment to the skull, and we simulate the natural modes of vibration. Direct measurements, which consisted of exciting vibration by tapping different spots across the TPC and recording the sound radiated in different directions, were performed on left and right TPCs still attached to the skull.

The vibrational measurements and modeling conducted in this study give insight into how the TPC may play a role in low frequency hearing. We can point to the pedicles and their characteristics and the material properties that affect their function, noting that the bulla can swing on them as if they were “door hinges” at the first few natural frequency modes (for example, in the lowest resonance mode S1 and S2 Videos). For higher frequency modes the displacement patterns tend to be more complex, with many modes concentrating energy at the sigmoidal process and therefore the malleus and the ossicular chain. The model results were found to be consistent with the experimental results, validating the modelling technique for fin whales. This result suggests that these modelling techniques could also work for other mysticetes which are morphologically similar. Since we are dealing with a particularly complicated and integral component of the mysticete hearing mechanism, our work can put in place pieces of the validation to support models that incorporate the entire skull.

## Materials and methods

### Fin whale skull

The skull used for this study is from an adult male fin whale (*Balaenoptera physalus*) with a straight length of 17.25 m, as recorded in the Marine Mammal Stranding Report Level A Data (Field Number :SWFSC-KXD0249, National Database Number :SW-2014–1157143) of May 19, 2014. The whale was first spotted floating, freshly deceased, by the Wastewater Treatment Plant at Point Loma in San Diego County, CA. The head was collected by Ted Cranford (SDSU) and transported to Camp Elliot Field Station. The head rested on the surface of the ground, covered with a black plastic tarp allowing dermestid beetles, also known as “skin beetles,” to clean the soft tissues from the skeletal elements. Following this, the skull was kept at Camp Elliot covered by a tarp. The skull was wrapped in chain-link fencing to hold the bones together during and after transport.

**Fig 1:**
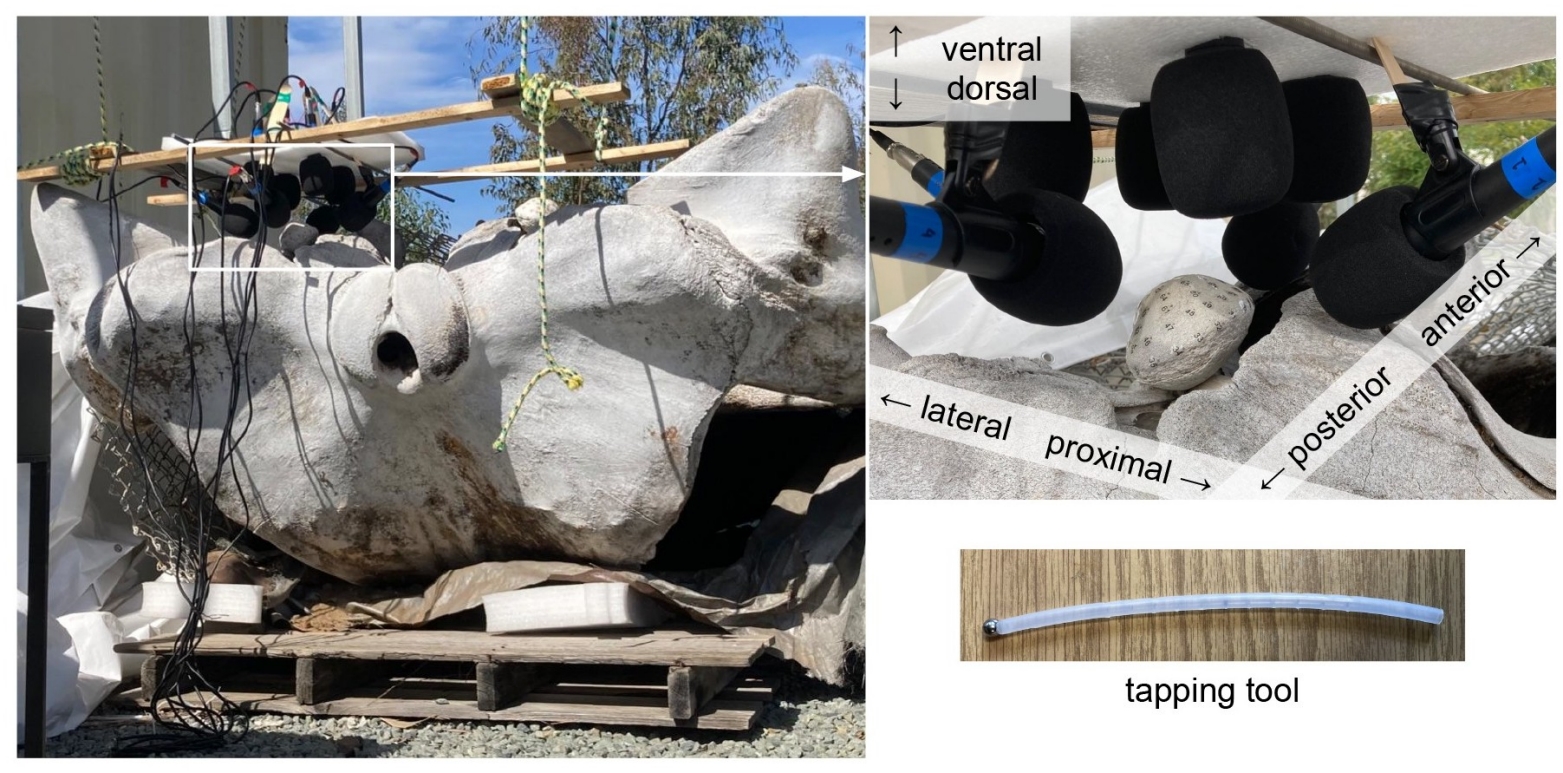
Recording arrangement. The fin whale skull rests ventral-side up with both left and right TPC exposed (left). Each TPC is covered in stickers numbered 1–89 with like numbers in comparable places on both TPCs. An arrangement of 8 microphones, labelled 1–8, is suspended over one TPC at a time (top right). Microphones are connected to a *MOTU 8pre* (out of frame) to facilitate simultaneous recording using the *Audacity* app for macOS. The tapping tool used was a 6mm diameter steel sphere adhered by silicone onto the end of a polyurethane tube (bottom right).

Both left and right TPC were attached to the fin whale skull. At the time of the experiment, the skull rested atop foam padding, ventral-side up so both TPC were exposed (Fig 1). The incus and malleus were present on both sides, but the ligament connecting them was absent. We were unable to determine whether the stapes was in place.

### TPC vibration measurement

We measured the left and right TPC separately, using the following process. Eight *Pyle PDMIC58* microphones were positioned around the tympanic bulla, arranged on a frame as shown in Fig 1. The microphones were separated and held in place by a slab of foam to prevent noise caused by resonance of a rigid frame. The microphone setup was suspended above the ventral-side-up skull to prevent any interaction between the frame and the vibrations of the TPC bones. Four microphones were positioned to face the anterior, posterior, lateral, and proximal sides of the TPC, while the remaining four were arranged in a plane directed towards the ventral side of the TPC. All eight microphones were connected to a *MOTU 8pre* for simultaneous recording using the *Audacity* app for macOS.

**Fig 2:**
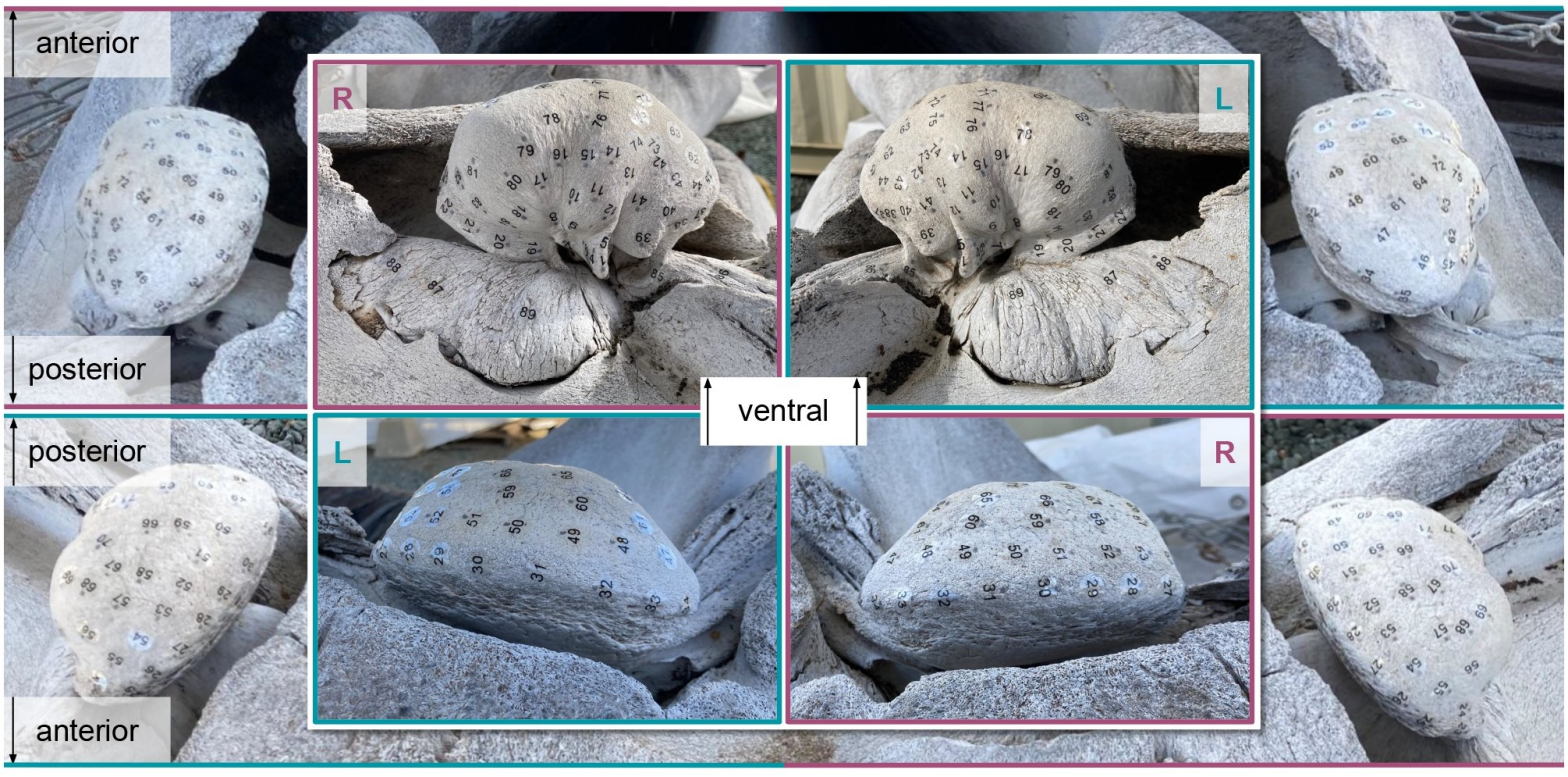
TPC tap locations. Tap locations denoted by stickers numbered 1–89 placed with like numbers in visually-comparable places on both TPCs. Tap locations are on the bone, just beyond the gray dot at the top of each sticker. Top Left: Right TPC posterior and lateral views. Top Right: Left TPC lateral and posterior views. Bottom Left: Left TPC anterior and proximal views. Bottom Right: Right TPC proximal and anterior views. All views are shown ventral-side-up, as this was the skull orientation during the experiment. The authors note that the stickers may not be in perfectly homologous locations; however, coverage over each TPC is comparable and TPC-scale vibration patterns are captured by both arrangements.

Numbered stickers were used to designate tap locations in visually comparable places on both left and right TPCs, with a higher concentration on and near the sigmoid process. A total of 89 tap locations were designated: Eighty-five on the tympanic bulla, one on the anterior pedicle, and three on the periotic bone (Fig 2). The posterior pedicle was excluded from tapping because it was too difficult to reach. At each tap location, we initiated a new recording, then used the tapping tool (Fig 1) to strike the bone at least 20 times with a short pause between each tap. Recordings from each tap location were exported as wav format files to be processed in *MATLAB*. We computed the power spectrum of the coherent sum of 20 taps from each tap location, and normalized the spectra at 15kHz.

The tapping-tool used was a 6mm steel sphere affixed to the end of a short polyurethane tube by silicone gel. To alleviate any concern about the effect of the tapping tool itself on the experimental results, we repeated taps at 3 tap locations with a variety of potential tapping tools: (1) a straight metal pick, (2) a steel nut jammed on the end of a short polyurethane tube, (3) a metal BB affixed to a thin, pliable plastic rod by silicone gel, and (4) a 3mm diameter sphere affixed to a medical lancet device by silicone gel. Each chosen location was tapped with each device 20 times: 10 times with light force, and 10 times with moderate-high force, with the exception of the modified lancet with which 10 taps total were performed. While the strength of force is subjective, the same human did all of these taps and judged that the ‘light’ and ‘heavy’ force taps would be at the extreme ends of the taps performed during the full experiment. We found that the nut (2) gave the most consistent result, likely due to its mass. Due to concerns about the geometry of the nut, it was replaced by a solid steel sphere of comparable mass in the final tapping tool.

### TPC vibroacoustic model

We modeled the natural mode vibrations of the tympanic bone and ossicular chain with a finite element model developed using the programming language *Julia* [7, 8]. A 3D mesh of the fin whale TPCs were generated from a CT scan. The fin whale CT scan was from a calf (NMNH 571562) discovered at the Smithsonian Institution without further stranding data. The CT images of the left TPC showed that the stapes was out of place. The stapes was consequently not included in the simulation mesh of the left TPC. The right TPC simulation included the entire ossicular chain. However, we were able to remove elements of the ossicular chain in some of the simulated meshes to compare differences between the left and right sides having the same anatomical elements in place.

Because the mesh included just the TPC, the base of the periotic bone was fixed with zero displacement, since it is embedded in the skull, and the rest of the model was allowed to move relative to the periotic bone’s attachment to the skull. Output of the models included the frequencies of natural vibration, and the relative displacements across the bone occurring for each vibrational mode. Physical properties of the bone are shown in Table 1 and were chosen based on the literature [9–13].

**Table 1:** Material properties used in the models.

	Stiff Bone	Flexible Bone	Stiff Ligament	Flexible Ligament
Density, *ρ* [kg/m^3^]	2300, 2400, 2500	2000	1200	1200
Young’s Modulus, *E* [GPa]	10, 15, 20, 25	5, 7, 9	0.001	0.001
Poisson’s ratio, *ν*	0.3	0.3	0.3	0.45

Hard bone includes the periotic and tympanic bone, malleus, incus, and stapes. Soft bone describes the anterior and posterior pedicles. The malleolar incudal ligament and incudostapedial ligament are set to stiff, and the annular ligament is flexible. Where multiple values are included, separate simulations were run with each of those values.

## Results

**Fig 3:**
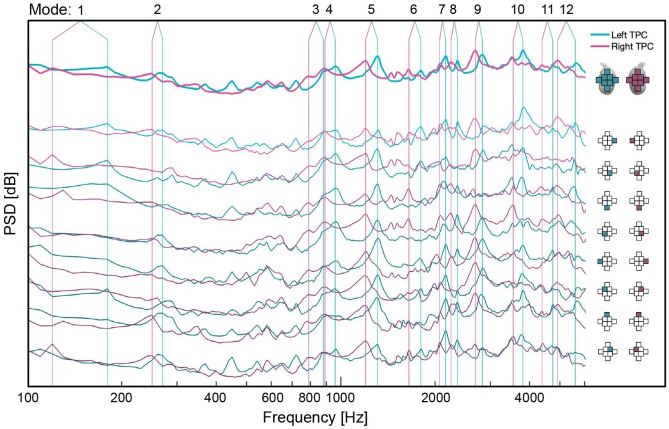
Spectra averaged across all tap locations. Spectra for the left and right sides are shown in blue and red respectively. The average across all microphones is shown at the top in bold. Spectra are displayed offset below for each microphone. The right panel indicates the position of the microphone relative to the TPC shown at the top of the panel. Vertical lines indicate the peaks identified as the first 12 resonance modes present in all simulations with or without the incus and stapes included.

Tapping the TPC resulted in the excitation of a set of well-defined resonance peaks within the frequency range of 100Hz to 6kHz. The overall pattern of resonant peaks was similar for all the microphones, although there were differences in the strength of the recorded sound related to the position of the microphone and the tap location. We present the average spectra over all tap locations for each microphone, as well as the average over all microphones (Fig 3). From the spectra, we identified resonance frequencies corresponding to the first 12 simulation modes (described below). Peaks that were identified as resonance modes appeared to some extent on all microphones for both TPC. For all modes, the left TPC produced a higher-frequency peak than the right TPC.

Our simulations yielded the mode shapes and corresponding frequencies for the TPCs at various Young’s Modulus and density values. To establish a correlation between our experiment and these simulations, we compared the recorded sound amplitudes with the normal component of the simulated displacement. The strength of the resonant motion normal to the bone surface should correspond with how well a particular resonant mode is excited by tapping along the normal to the surface at that specific location. We identified the normal component of the simulated displacement to determine how each mode responds to tapping, and compared it with the recorded amplitudes.

The simulations revealed resonance modes involving the whole TPC and others exhibiting more localized vibration in the ossicular chain, with the tympanic bulla remaining stationary. In the physical experiment, the malleolar-incudal ligament was absent, so we would not expect the latter of those modes to be excited. When the simulation was run with the incus and stapes removed, these resonances disappeared as expected, while the others persisted. For these modes, the vibrations across the tympanic bulla and pedicles remained relatively unchanged with or without the ossicular chain present in the simulation, yet they still resulted in movements of the malleus, which, in turn drives motions through the ossicular chain. The resonance frequencies and mode shapes were only slightly altered. We focused on the first 12 vibration modes present with and without the ossicular chain included in the simulation, referring to them as frequency modes 1–12.

**Fig 4:**
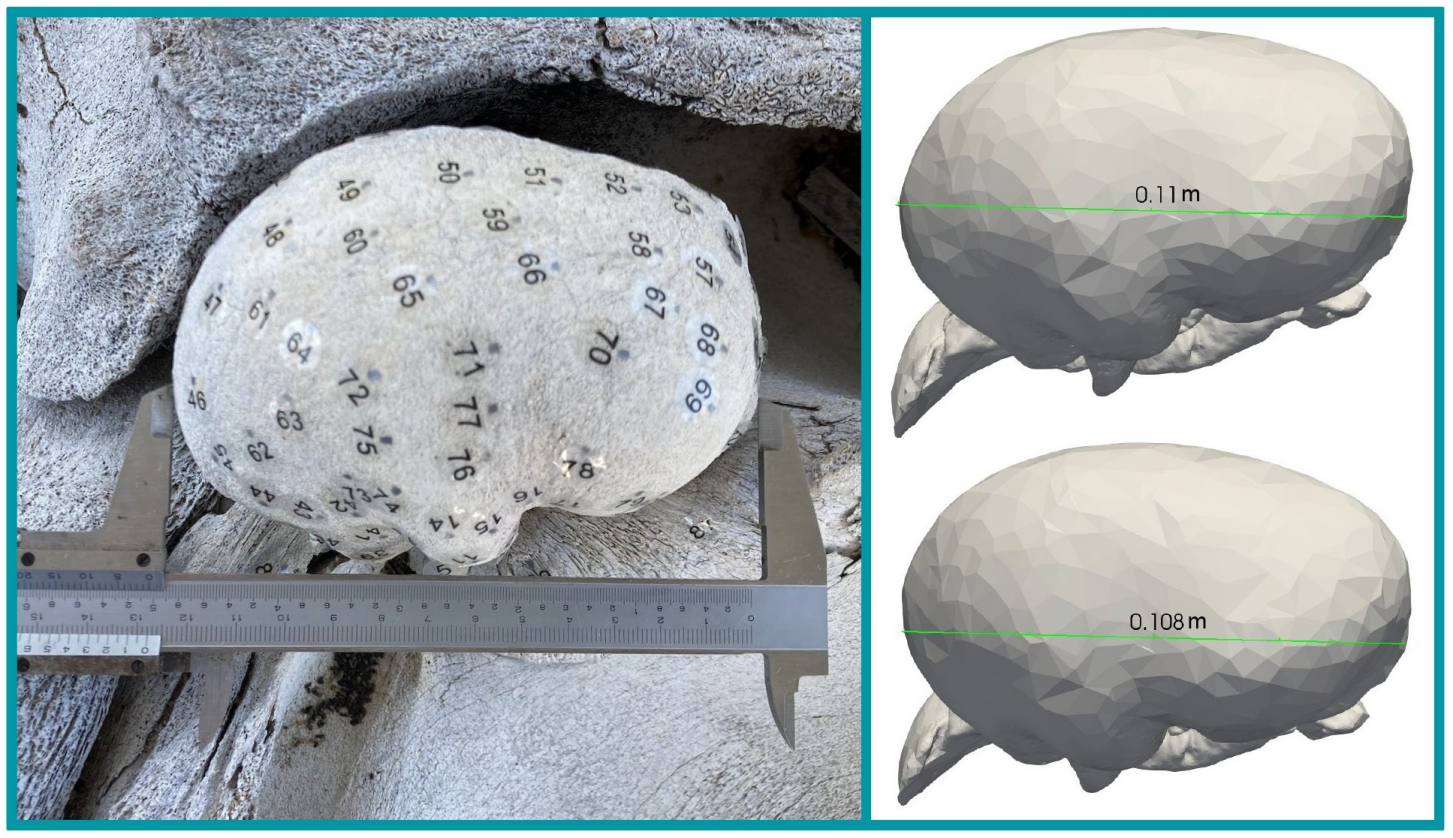
Size comparison between left TPC of the experiment and the simulation. Left: Left TPC of the experiment with calipers measuring 12.8cm TPC ‘length’. Right: Left TPC mesh with two alternate ‘length’ measurements. The top and bottom measurements are the longest and shortest reasonable ‘length’ measurement that could correspond to the dimension measured in the left panel. Although it is imprecise to compare sizes based on a single measurement, the comparison is meant to demonstrate that the TPCs in the experiment are larger than those in the simulation.

We also ran the simulation with the TPC mesh uniformly scaled up because the tapped TPCs have larger dimension than the simulated TPCs (Fig 4). Measurements of the ratio between the tapped TPCs and the simulated TPCs varied from 1.16 to 1.19. We scaled the mesh by a factor of 1.2, which we chose as an upper-bound for the size difference. The simulated mode shapes were nearly identical between the scaled and original meshes. Frequencies were lower by a factor of about 1.2 for the scaled mesh.

The simulations varied the density for the stiff bone and the Young’s moduli for both stiff and flexible bone (Table 1). Increasing the Young’s modulus generally resulted in a higher resonance frequency, while increasing the density led to a lower resonance frequency. Modes 1–3 and 7–10 exhibited consistent modes shapes, but the order of modes 4–6 changed when Young’s modulus was altered. Specifically, modes 4, 5, 6 with Young’s modulus for stiff and flexible bone set to 25 and 5 GPa respectively, correspond to modes 6, 4, 5 when Young’s modulus for stiff and flexible bone were set to 10 and 9 GPa. We found that the modes identified in the experiment most closely resembled the order found with Young’s modulus for stiff and flexible bone set to 25 and 5 GPa.

**Fig 5:**
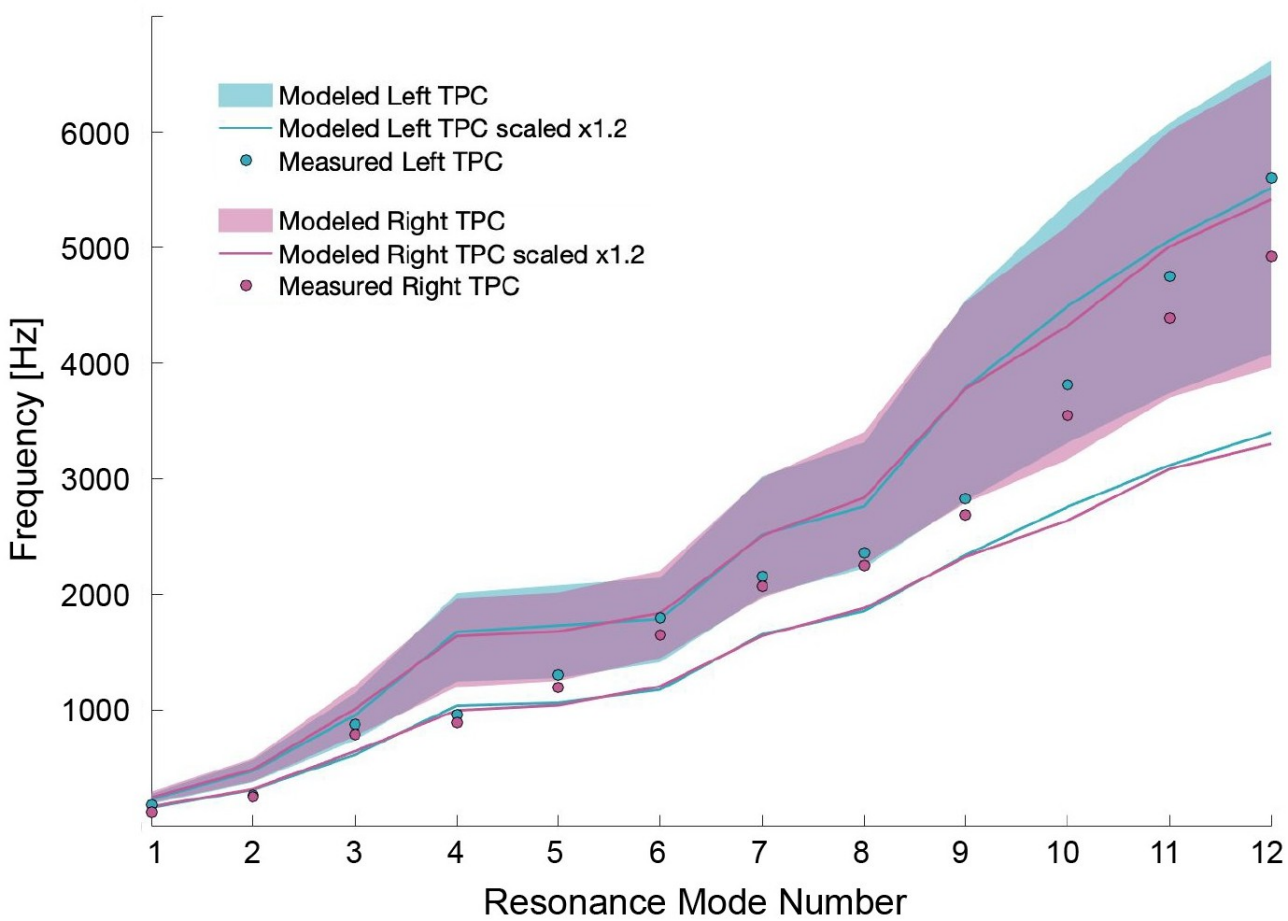
Modeled and measured resonance frequencies for modes 1–12. Resonance frequencies identified in the measured spectra are shown as blue and red dots for the left and right TPC respectively. The shaded region shows the range of resonance frequencies obtained through the model over all material properties used. The outlined region shows the range of resonance frequencies over all material parameters with the TPC uniformly scaled by a factor of 1.2 (approximate size difference between the modeled and measured TPC).

**Fig 6:**
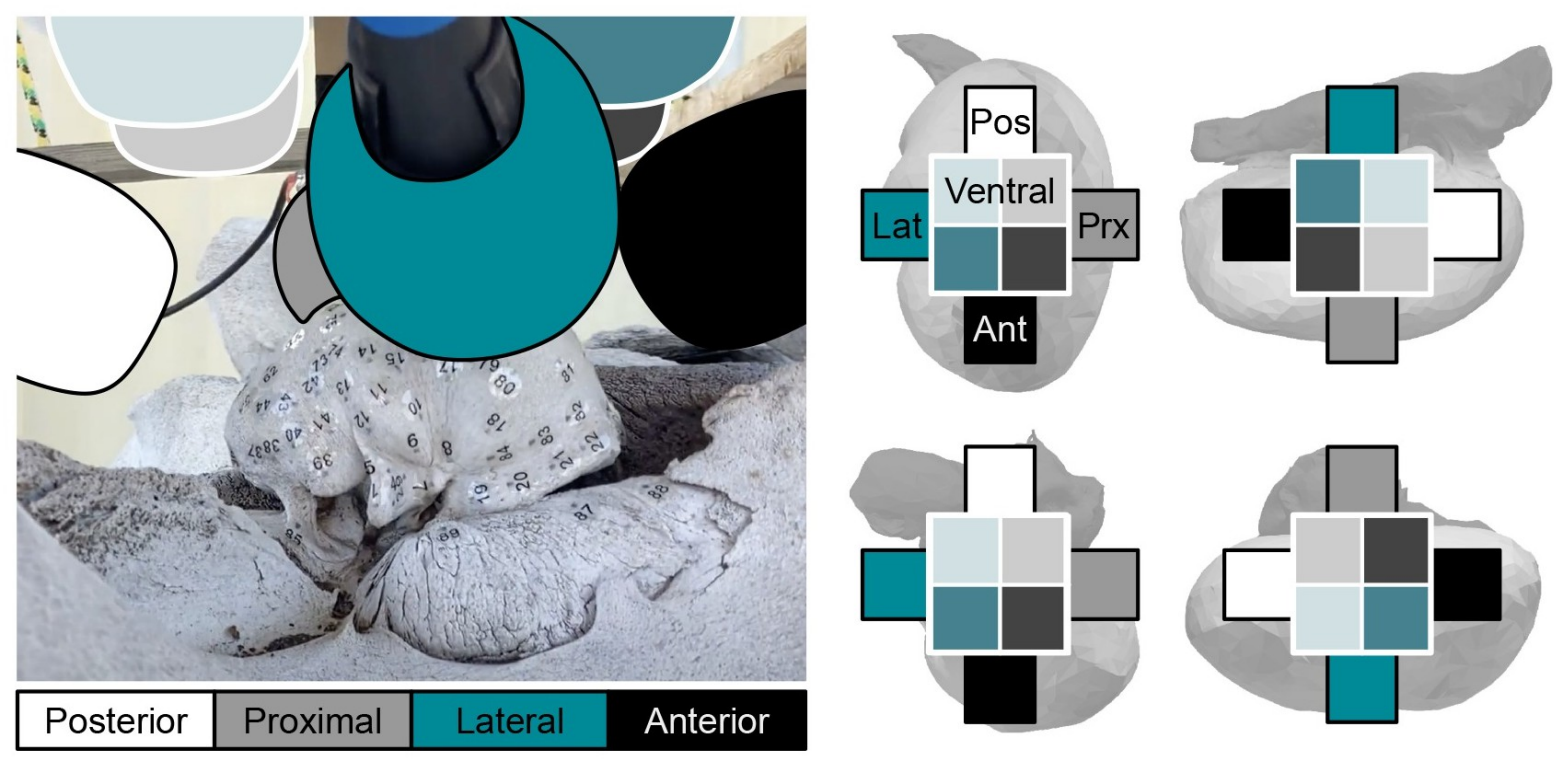
Microphone arrangement key for mode comparison plots. In the mode comparison figures (Figs 7–9, and supplemental), received amplitude from each microphone is shown in a ‘plus’ formation that mimics the arrangement of the eight microphones over each panel. This figure describes which the squares in the ‘plus’ correspond to which microphone for each orientation, using the Left TPC as the example. On the right, the ‘plus’ shape is enlarged over each TPC orientation for clarity. Boxes representing each microphone are colored to match the corresponding microphone color in the left image. In the upper left panel of the left TPC, for example: the top, right, bottom, and left of the ‘plus’ represents the posterior, proximal, anterior, and lateral microphones respectively. The interior squares on the ‘plus’ represent the ventral microphones.

**Fig 7:**
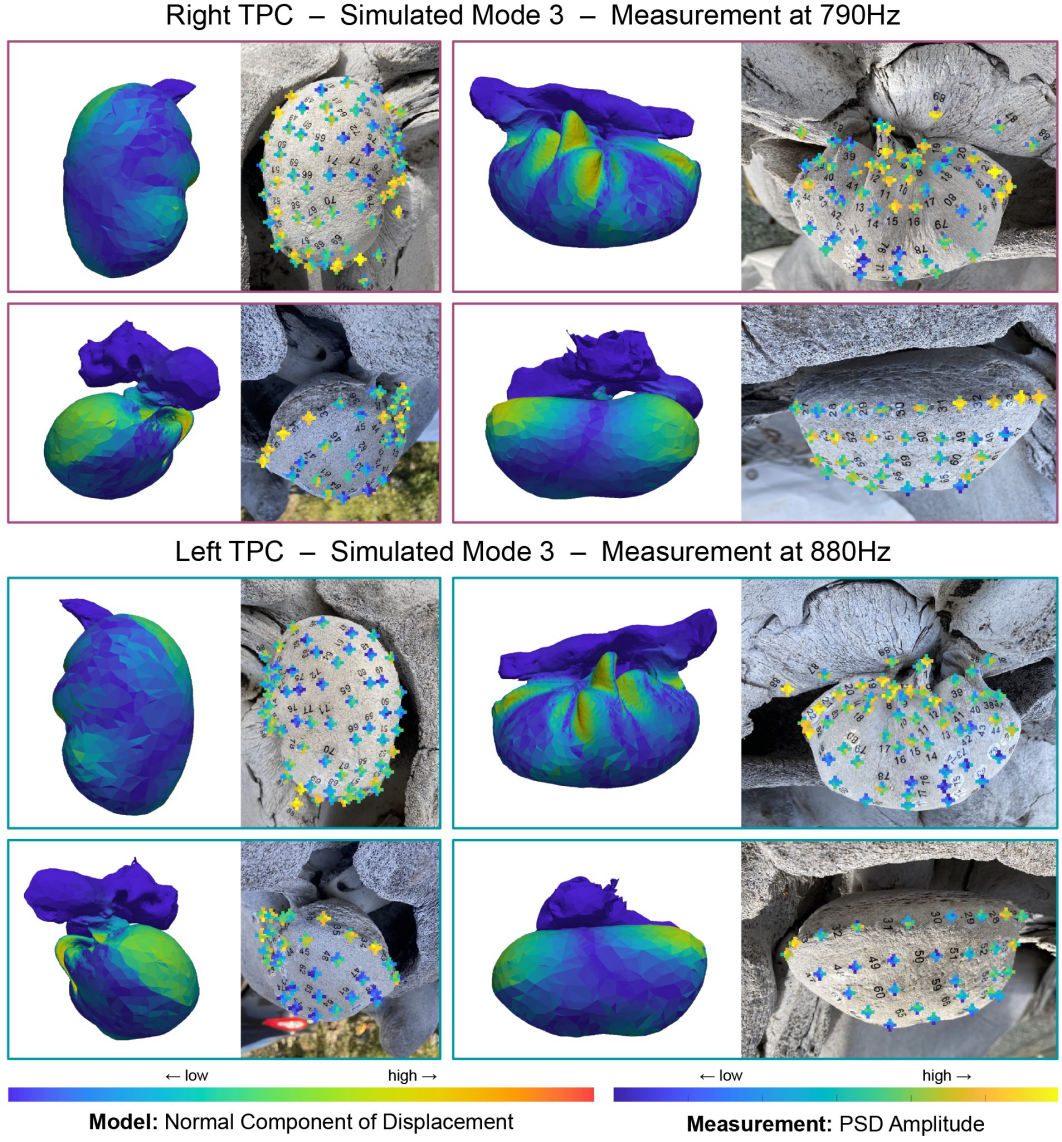
Comparison of simulated mode 3 with measurements of the left TPC at 880Hz and the right at 790Hz. Left on each panel shows the amplitude of the normal component of displacement for the simulated TPC mode 3 (stiff bone: *ρ* = 2400kg/m^3^, *E* = 25GPa, flexible bone: *ρ* = 2000kg/m^3^, *E* = 5GPa). Right on each panel shows an image of the TPC with received amplitudes at 880Hz or 790Hz overlaid as color. At each tap location, the received amplitude from each microphone is shown in a ‘plus’ formation (Fig 6). Both model and simulation show highest amplitudes on the posterior side of the sigmoid process, on the anterior and posterior edges of the proximal surface, and on the anterior lip. Relatively high amplitudes are registered by the anterior and posterior microphones on most tap locations. Note that the left and right TPC exhibit this pattern at different frequencies.

**Fig 8:**
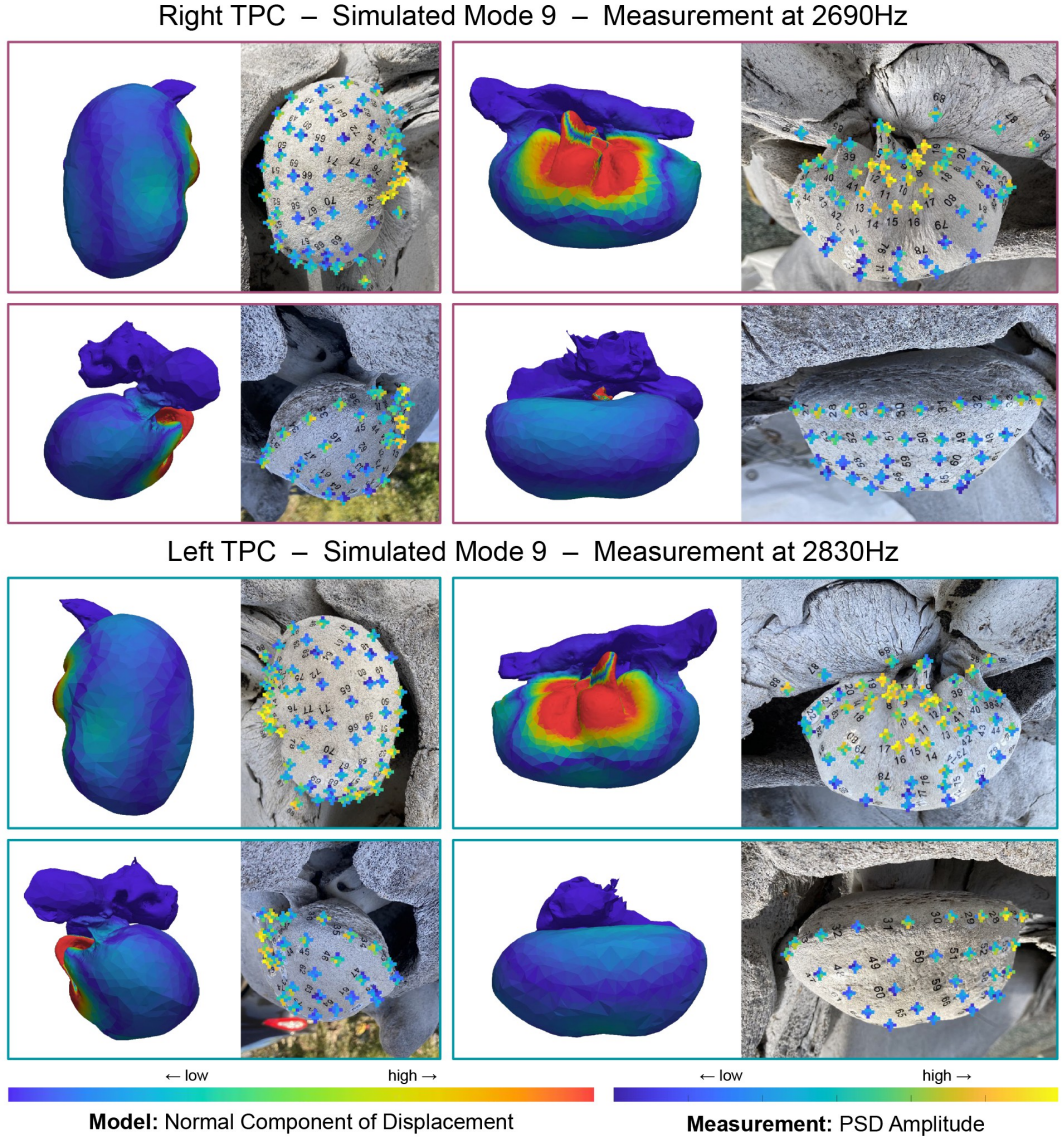
Comparison of simulated mode 9 with measurements of the left TPC at 2830Hz and the right at 2690Hz. Left on each panel shows the amplitude of the normal component of displacement for the simulated TPC mode 9 (stiff bone: *ρ* = 2400kg/m^3^, *E* = 25GPa, flexible bone: *ρ* = 2000kg/m^3^, *E* = 5GPa). Right on each panel shows an image of the TPC with received amplitudes at 2830Hz or 2690Hz overlaid as color. At each tap location, the received amplitude from each microphone is shown in a ‘plus’ formation (Fig 6). For this mode, taps on and very near the sigmoid process generate the highest amplitudes. Additionally, higher amplitudes are received by the lateral and anterior microphones for most tapped locations. Simulated mode 9 shows higher displacement also concentrated on the lateral side of the TPC. Both simulated and measured modes show a node along the sigmoid process. Note that the left and right TPC exhibit this pattern at different frequencies.

**Fig 9:**
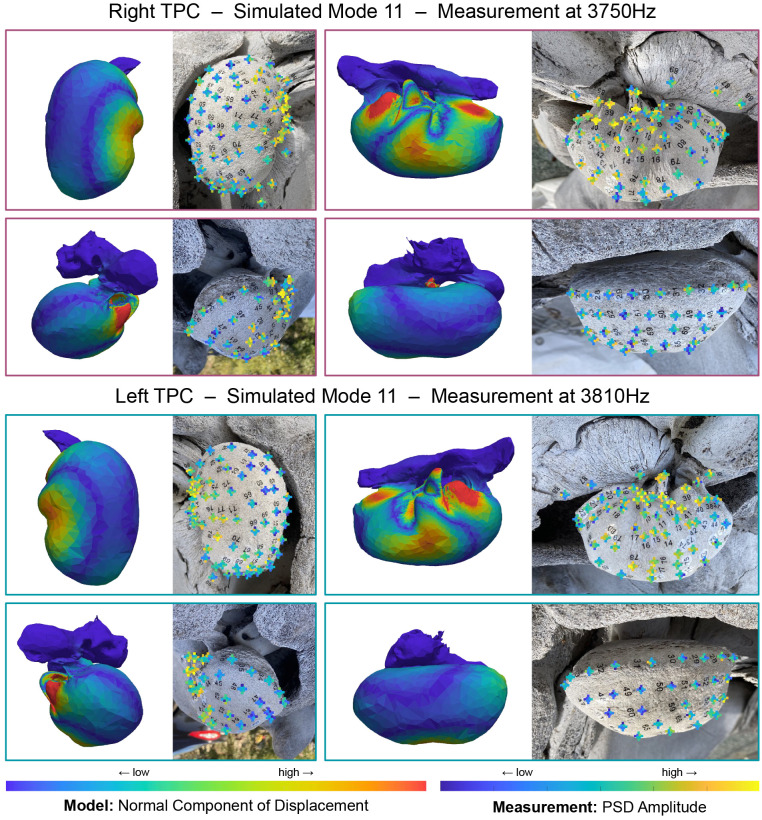
Comparison of simulated mode 11 with measurements of the left TPC at 3810Hz and the right at 3750Hz. Left on each panel shows the amplitude of the normal component of displacement for the simulated TPC mode 11 (stiff bone: *ρ* = 2400kg/m^3^, *E* = 25GPa, flexible bone: *ρ* = 2000kg/m^3^, *E* = 5GPa). Right on each panel shows an image of the TPC with received amplitudes at 3810Hz or 3750Hz overlaid as color. At each tap location, the received amplitude from each microphone is shown in a ‘plus’ formation (Fig 6). Measured modes show high amplitudes received from the conical process and lateral-ventral tap locations, which also show high normal-displacement in the simulations. Arching nodes can be seen on the proximal side in all cases. Note that the left and right TPC exhibit this pattern at different frequencies.

The modeled resonance frequencies are in good agreement with the measured frequencies when the modeled TPC is uniformly scaled by a factor up to 1.2, the approximate size difference between the modeled and measured TPCs (Fig 5). We also show the amplitude of the received sound at each microphone for select frequencies, displayed for each tap location on an image of the tapped-TPC (Fig 6). Specifically, Figs 7–9 show modes 3, 9, and 11 respectively. All mode comparisons are included S1–S24 Figs. Our analysis revealed that the pattern of tap locations resulting in the highest amplitude of radiated sound correspond to regions of the model with high amplitude of vibration normal to the bone. In those high-amplitude regions, sound is radiated with a pattern that results from the motion across the bulla.

## Discussion

We observed remarkable similarities between the experiment and simulated mode shapes and corresponding frequencies, despite some notable differences between the modeled and measured TPCs, including the attachment to the skull, ligament condition, and bone geometry. We first discuss these differences in more detail. Subsequently, we consider our findings related to fin whale hearing, based on the results obtained from our experiments and simulations.

The first major difference between the modeled and measured TPC is that the latter is attached to the skull, while the model contains only the TPC. To account for this, we simulated resonance modes of the TPC with the base of the periotic bone fixed in place, as it would be attached to the skull. This simulates a firm attachment to the skull, resulting in modes of resonance relative to the skull.

Another notable difference between the modeled and the measured TPC is the inclusion of the ligaments in the ossicular chain in the former. In the experiment, the malleolar-incudal ligament was absent, so the ossicular chain was not well-connected. Consequently, we would not expect modes primarily involving the ossicular chain to be excited. Nevertheless, we found that modes involving vibration across the entire TPC were present in the simulation, regardless of whether the ossicular chain was included or not. Therefore, despite the absence of the ossicular chain’s ligaments in the experiment, modes that should be present when everything is connected were still excited, providing insight into the mechanisms underlying fin whale hearing.

Another consideration is that since the TPC in the experiment was not the same one scanned for the model, there are bound to be some discrepancies between model and experiment resulting from true differences in geometry. One clear difference is in size: the length of the physical adult tympanic bulla in the experiment is at most 1.2 times that of the calf TPC in the model (Fig 4). Scaling-up the mesh resulted in lower resonance frequencies for the same vibration pattern, which could explain why the resonance frequencies identified in the experiment were lower than those in the model. However, a uniform scaling of the mesh cannot resolve any morphological discrepancies, and that it may overestimate bone thickness, leading to a further overestimation in frequency. It is important to consider these limitations when comparing the measured and modeled frequencies, yet Fig 5 shows that the measured frequencies are fairly consistent with the range of modeled frequencies obtained by varying material parameters, whether the TPC is scaled or not.

The model results depended upon the material parameters, including density and Young’s modulus, as well as the mesh geometry. We examined sensitivity of the model to changes in the Young’s modulus of both hard and soft bone, as well as the density of the hard bone. The model classified the pedicles as soft bone, while the rest of the TPC, including the ossicles, was classified as hard bone.

Our results show that density changes within the range reported in the literature have little effect on the mode shape. However, increasing the density of the hard bone leads to a proportional decrease in resonant frequency for each mode, with a 4% increase in density corresponding to a 4% decrease in frequency for each mode. On the other hand, changing the Young’s modulus has a larger affect on the mode shapes. As the true Young’s modulus of the bone is uncertain, the range of values that we considered reasonable for the model inputs led to a wider range of possible frequencies and possible modes. The elastic moduli measured by Tubelli et al 2004 [10] for fin whale ossicles are broad, and may be considered an upper bound, given that bone is porous, and the nanoindentation method they used was by definition applied to the solid framework of the bone. Additionally, the skull used in the experiment had been dried for some time, and studies have shown that Young’s modulus may increase for dried bone [14]. However, since the TPC bone is dense, the effect of drying is expected to be less pronounced than in other types of bone. Nonetheless, the wide range of potentially appropriate Young’s moduli leads to some variation in mode shapes and frequencies.

Several modes are clearly identifiable in both the simulation and experiment, such as mode 9, shown in Fig 8, where higher amplitudes are concentrated in a region around the sigmoidal process and lateral furrow of the tympanic bulla. In the simulation, a “node” on the sigmoidal process (the blue strip where the normal component of displacement is almost zero) corresponds to tap locations that produce lower amplitudes. Additionally, higher amplitudes are received by the lateral and anterior microphones for most tap locations in the high-amplitude region. Simulated mode 9 exhibits more deformation on the lateral and anterior sides of the bulla. This can be seen in the displacement pattern, and is made clear in an animation of this mode (S5 and S6 Videos).

However, some modes are more difficult to distinguish, which may be due to the nature of the experiment. The simulation shows each mode as distinct from one another, displaying how the bone would vibrate if driven at each resonance frequency alone, moving solely in its mode. In reality, the motion of the TPC is often a combination of multiple modes. Mathematically, the vibration of any object can be described as a sum of that object’s normal modes of vibration. During the experiment, multiple modes are excited simultaneously, and by taking the FFT and producing the power spectrum, we are decomposing the recording into its frequency components to isolate the motion at specific frequencies. This has the effect that when we measure the recorded amplitude at frequencies between resonance modes, the spatial pattern will likely show a combination of elements from adjacent (and possibly more) resonant modes.

Notably, if two modes are close enough in frequency, it will be difficult to separate them in the data. This can be seen in the recorded spectra most clearly for frequencies between 800–1000Hz (Fig 3), where the left (and to a lesser degree the right) TPC produces two peaks, identified as modes 3 and 4, that nearly appear as one. If the resonance frequencies were closer, it would be challenging to resolve the difference in the experimental data, while in the simulation both modes would be shown as separate and distinct. These factors may explain some of the discrepancies between the modeled and measured modal patterns.

For instance, mode 3 (Fig 7) shows fairly good agreement between high-amplitude tap locations and simulated regions with high normal displacement, but one discrepancy is that the node on the proximal side of the bulla is not as pronounced in the experiment as it is in the simulation. That node is not present in simulated mode 4 (S4 and S16 Figs), which may be contributing to the displacement pattern resolved in the experiment. This can also be seen in the radiated sound pattern recorded by the microphones. The bulla in the simulated mode is swishing anterior to posterior with a slight pivot along the aforementioned proximal node (S3 and S4 Videos), so we would expect sound to radiate in the anterior and posterior directions with relatively little sound radiated proximally. The anterior and posterior microphones record high amplitudes as expected, but the proximal microphone also records relatively high amplitudes for many tap-locations. Again, this unexpected sound may be from mode 4, whose frequency peak broadly overlaps with mode 3.

As frequency increases, vibration patterns become more complex and the modes become difficult to resolve. Mode 11 (Fig 9) provides an example where it is possible to identify correlations between the simulation and experiment, but the match is not as clear as that in mode 9 (Fig 8). Mode 11 shows high amplitudes on the conical process and lateral-ventral tap locations in both simulation and experiment, and exhibits an arching node across the proximal side. However, the high amplitudes simulated on the anterior lip are absent from the experiment. Vibrations across the bulla are complex (S7 and S8 Videos), so it is more difficult to imagine what its radiation pattern should look like. Some tap locations anterior to the sigmoid process on the left TPC produce low amplitudes for the ventral microphones as may be expected by the node along the ventral side of the bulla. The radiation pattern seen by the microphones is less consistent across tap locations on the right TPC. This may in part be because modes 11 and 12 are closer in frequency for the right TPC than they are for the left.

While the modeled and measured TPCs were from different specimens, both were from fin whales with fully-developed TPCs, as the whale in the physical experiment was an adult and the CT scan was of a calf whose TPCs would have been developed precocially [15]. While there is natural variation between whales of the same species, we would expect substantial overlap in the frequency range that all can hear. Further, while resonances of the TPC itself may not be the same in all specimens, they should cover a similar range of frequencies to allow for communication. We identified multiple resonances between 100Hz and 6kHz in the experiment as well as in all modeled cases, with Young’s modulus of the bone ranging from 5–25GPa and density ranging from 2300–2500 kg/m^3^. Although higher resonances were recorded, the mode shapes became more difficult to identify in the measurements, so we do not present them here. Nevertheless, the consistency between modeled and measured mode shapes for modes under 6kHz gives us confidence in using the models to investigate the full range of fin whale hearing sensitivity. Most of the identified resonances concentrate the vibration near the sigmoidal process, driving vibrations in the ossicular chain, and thus playing a significant role in the whale’s hearing capabilities.

Moreover, the model highlights that lower frequency modes particularly involve considerable movement of the bulla swinging relative to the skull, with the pedicles acting as hinges (See mode 1 as an example S1 and S2 Videos) or exhibiting a twisting movement. Our previous work has also shown the TPC dancing on its pedicles in both mysticetes and odontocetes [3, 16]. This implies that the pedicles are a significant component of these motions that drive oscillations in the ossicular chain and are best kept intact in models of whale hearing.

One further observation that can be made from both model and measurement is that, for each mode, the resonance frequencies of the left and right TPCs are offset (Fig 3). Interestingly, in the measurements, the left TPC modes were found to have higher frequencies, whereas in the model, they were sometimes higher and sometimes lower. It is likely that it is not possible to build a perfectly symmetric bilateral organism, so this offset is inevitable. Asymmetry may play a role in the directionality of hearing, with both ears being sensitive to different frequencies. Structural asymmetry proves useful in resolving ambiguities in a symmetrical sound field in a number of bioacoustic examples including dolphins [17, 18], owls [19, 20], and some insects [21]. Moreover, this characteristic asymmetry likely contributes to improving hearing sensitivity across the range of frequencies that the whales can hear, as each TPC will be more sensitive to frequencies near its resonance modes, so it will be more sensitive at frequencies for which the other is less sensitive.

## Conclusion

We demonstrate that the natural modes of vibration of the fin whale tympanoperiotic complex (TPC) involve swinging or twisting on the suspensory pedicles, with many vibration modes concentrating energy at the sigmoidal process and therefore the malleus and the ossicular chain. Resonance frequencies range from 100Hz to beyond 6kHz, indicating that TPC resonance contributes to hearing sensitivity in that range. Our findings reveal that the resonance frequencies of the left and right TPC are offset, providing a potential mechanism for improved hearing at a range of frequencies as well as a mechanism for directionality in sound perception. Furthermore, our model results are in agreement with the experimental results, validating the modeling techniques that have been successfully employed for odontocetes and suggesting their applicability for mysticetes. Since we are dealing with a complex and integral part of the mysticete hearing mechanism, this should build confidence in future work using the models of entire skull vibrations.

## Supporting information

S1 Fig Comparison of simulated mode 1 with measurements of the right TPC at 120Hz. Left on each panel shows the amplitude of the normal component of displacement for the simulated TPC mode (stiff bone: *ρ* = 2400kg/m^3^, *E* = 25GPa, flexible bone: *ρ* = 2000kg/m^3^, *E* = 5GPa). Right on each panel shows an image of the TPC with received amplitudes overlaid as color.
(TIF)

S2 Fig Comparison of simulated mode 2 with measurements of the right TPC at 250Hz. Left on each panel shows the amplitude of the normal component of displacement for the simulated TPC mode (stiff bone: *ρ* = 2400kg/m^3^, *E* = 25GPa, flexible bone: *ρ* = 2000kg/m^3^, *E* = 5GPa). Right on each panel shows an image of the TPC with received amplitudes overlaid as color.
(TIF)

S3 Fig Comparison of simulated mode 3 with measurements of the right TPC at 790Hz. Left on each panel shows the amplitude of the normal component of displacement for the simulated TPC mode (stiff bone: *ρ* = 2400kg/m^3^, *E* = 25GPa, flexible bone: *ρ* = 2000kg/m^3^, *E* = 5GPa). Right on each panel shows an image of the TPC with received amplitudes overlaid as color.
(TIF)

S4 Fig Comparison of simulated mode 4 with measurements of the right TPC at 890Hz. Left on each panel shows the amplitude of the normal component of displacement for the simulated TPC mode (stiff bone: *ρ* = 2400kg/m^3^, *E* = 25GPa, flexible bone: *ρ* = 2000kg/m^3^, *E* = 5GPa). Right on each panel shows an image of the TPC with received amplitudes overlaid as color.
(TIF)

S5 Fig Comparison of simulated mode 5 with measurements of the right TPC at 1200Hz. Left on each panel shows the amplitude of the normal component of displacement for the simulated TPC mode (stiff bone: *ρ* = 2400kg/m^3^, *E* = 25GPa, flexible bone: *ρ* = 2000kg/m^3^, *E* = 5GPa). Right on each panel shows an image of the TPC with received amplitudes overlaid as color.
(TIF)

S6 Fig Comparison of simulated mode 6 with measurements of the right TPC at 1650Hz. Left on each panel shows the amplitude of the normal component of displacement for the simulated TPC mode (stiff bone: *ρ* = 2400kg/m^3^, *E* = 25GPa, flexible bone: *ρ* = 2000kg/m^3^, *E* = 5GPa). Right on each panel shows an image of the TPC with received amplitudes overlaid as color.
(TIF)

S7 Fig Comparison of simulated mode 7 with measurements of the right TPC at 2070Hz. Left on each panel shows the amplitude of the normal component of displacement for the simulated TPC mode (stiff bone: *ρ* = 2400kg/m^3^, *E* = 25GPa, flexible bone: *ρ* = 2000kg/m^3^, *E* = 5GPa). Right on each panel shows an image of the TPC with received amplitudes overlaid as color.
(TIF)

S8 Fig Comparison of simulated mode 8 with measurements of the right TPC at 2250Hz. Left on each panel shows the amplitude of the normal component of displacement for the simulated TPC mode (stiff bone: *ρ* = 2400kg/m^3^, *E* = 25GPa, flexible bone: *ρ* = 2000kg/m^3^, *E* = 5GPa). Right on each panel shows an image of the TPC with received amplitudes overlaid as color.
(TIF)

S9 Fig Comparison of simulated mode 9 with measurements of the right TPC at 2690Hz. Left on each panel shows the amplitude of the normal component of displacement for the simulated TPC mode (stiff bone: *ρ* = 2400kg/m^3^, *E* = 25GPa, flexible bone: *ρ* = 2000kg/m^3^, *E* = 5GPa). Right on each panel shows an image of the TPC with received amplitudes overlaid as color.
(TIF)

S10 Fig Comparison of simulated mode 10 with measurements of the right TPC at 3550Hz. Left on each panel shows the amplitude of the normal component of displacement for the simulated TPC mode (stiff bone: *ρ* = 2400kg/m^3^, *E* = 25GPa, flexible bone: *ρ* = 2000kg/m^3^, *E* = 5GPa). Right on each panel shows an image of the TPC with received amplitudes overlaid as color.
(TIF)

S11 Fig Comparison of simulated mode 11 with measurements of the right TPC at 3750Hz. Left on each panel shows the amplitude of the normal component of displacement for the simulated TPC mode (stiff bone: *ρ* = 2400kg/m^3^, *E* = 25GPa, flexible bone: *ρ* = 2000kg/m^3^, *E* = 5GPa). Right on each panel shows an image of the TPC with received amplitudes overlaid as color.
(TIF)

S12 Fig Comparison of simulated mode 12 with measurements of the right TPC at 4920Hz. Left on each panel shows the amplitude of the normal component of displacement for the simulated TPC mode (stiff bone: *ρ* = 2400kg/m^3^, *E* = 25GPa, flexible bone: *ρ* = 2000kg/m^3^, *E* = 5GPa). Right on each panel shows an image of the TPC with received amplitudes overlaid as color.
(TIF)

S13 Fig Comparison of simulated mode 1 with measurements of the left TPC at 180Hz. Left on each panel shows the amplitude of the normal component of displacement for the simulated TPC mode (stiff bone: *ρ* = 2400kg/m^3^, *E* = 25GPa, flexible bone: *ρ* = 2000kg/m^3^, *E* = 5GPa). Right on each panel shows an image of the TPC with received amplitudes overlaid as color.
(TIF)

S14 Fig Comparison of simulated mode 2 with measurements of the left TPC at 270Hz. Left on each panel shows the amplitude of the normal component of displacement for the simulated TPC mode (stiff bone: *ρ* = 2400kg/m^3^, *E* = 25GPa, flexible bone: *ρ* = 2000kg/m^3^, *E* = 5GPa). Right on each panel shows an image of the TPC with received amplitudes overlaid as color.
(TIF)

S15 Fig Comparison of simulated mode 3 with measurements of the left TPC at 880Hz. Left on each panel shows the amplitude of the normal component of displacement for the simulated TPC mode (stiff bone: *ρ* = 2400kg/m^3^, *E* = 25GPa, flexible bone: *ρ* = 2000kg/m^3^, *E* = 5GPa). Right on each panel shows an image of the TPC with received amplitudes overlaid as color.
(TIF)

S16 Fig Comparison of simulated mode 4 with measurements of the left TPC at 960Hz. Left on each panel shows the amplitude of the normal component of displacement for the simulated TPC mode (stiff bone: *ρ* = 2400kg/m^3^, *E* = 25GPa, flexible bone: *ρ* = 2000kg/m^3^, *E* = 5GPa). Right on each panel shows an image of the TPC with received amplitudes overlaid as color.
(TIF)

S17 Fig Comparison of simulated mode 5 with measurements of the left TPC at 1310Hz. Left on each panel shows the amplitude of the normal component of displacement for the simulated TPC mode (stiff bone: *ρ* = 2400kg/m^3^, *E* = 25GPa, flexible bone: *ρ* = 2000kg/m^3^, *E* = 5GPa). Right on each panel shows an image of the TPC with received amplitudes overlaid as color.
(TIF)

S18 Fig Comparison of simulated mode 6 with measurements of the left TPC at 1800Hz. Left on each panel shows the amplitude of the normal component of displacement for the simulated TPC mode (stiff bone: *ρ* = 2400kg/m^3^, *E* = 25GPa, flexible bone: *ρ* = 2000kg/m^3^, *E* = 5GPa). Right on each panel shows an image of the TPC with received amplitudes overlaid as color.
(TIF)

S19 Fig Comparison of simulated mode 7 with measurements of the left TPC at 2160Hz. Left on each panel shows the amplitude of the normal component of displacement for the simulated TPC mode (stiff bone: *ρ* = 2400kg/m^3^, *E* = 25GPa, flexible bone: *ρ* = 2000kg/m^3^, *E* = 5GPa). Right on each panel shows an image of the TPC with received amplitudes overlaid as color.
(TIF)

S20 Fig Comparison of simulated mode 8 with measurements of the left TPC at 2360Hz. Left on each panel shows the amplitude of the normal component of displacement for the simulated TPC mode (stiff bone: *ρ* = 2400kg/m^3^, *E* = 25GPa, flexible bone: *ρ* = 2000kg/m^3^, *E* = 5GPa). Right on each panel shows an image of the TPC with received amplitudes overlaid as color.
(TIF)

S21 Fig Comparison of simulated mode 9 with measurements of the left TPC at 2830Hz. Left on each panel shows the amplitude of the normal component of displacement for the simulated TPC mode (stiff bone: *ρ* = 2400kg/m^3^, *E* = 25GPa, flexible bone: *ρ* = 2000kg/m^3^, *E* = 5GPa). Right on each panel shows an image of the TPC with received amplitudes overlaid as color.
(TIF)

S22 Fig Comparison of simulated mode 10 with measurements of the left TPC at 3810Hz. Left on each panel shows the amplitude of the normal component of displacement for the simulated TPC mode (stiff bone: *ρ* = 2400kg/m^3^, *E* = 25GPa, flexible bone: *ρ* = 2000kg/m^3^, *E* = 5GPa). Right on each panel shows an image of the TPC with received amplitudes overlaid as color.
(TIF)

S23 Fig Comparison of simulated mode 11 with measurements of the left TPC at 3810Hz. Left on each panel shows the amplitude of the normal component of displacement for the simulated TPC mode (stiff bone: *ρ* = 2400kg/m^3^, *E* = 25GPa, flexible bone: *ρ* = 2000kg/m^3^, *E* = 5GPa). Right on each panel shows an image of the TPC with received amplitudes overlaid as color.
(TIF)

S24 Fig Comparison of simulated mode 12 with measurements of the left TPC at 5600Hz. Left on each panel shows the amplitude of the normal component of displacement for the simulated TPC mode (stiff bone: *ρ* = 2400kg/m^3^, *E* = 25GPa, flexible bone: *ρ* = 2000kg/m^3^, *E* = 5GPa). Right on each panel shows an image of the TPC with received amplitudes overlaid as color.
(TIF)

S1 Video Simulated Mode 1 of the right TPC. Animation of simulated TPC mode colored by the normal component of displacement (stiff bone: *ρ* = 2400kg/m^3^, *E* = 25GPa, flexible bone: *ρ* = 2000kg/m^3^, *E* = 5GPa).
(MP4)

S2 Video Simulated Mode 1 of the left TPC. Animation of simulated TPC mode colored by the normal component of displacement (stiff bone: *ρ* = 2400kg/m^3^, *E* = 25GPa, flexible bone: *ρ* = 2000kg/m^3^, *E* = 5GPa).
(MP4)

S3 Video Simulated Mode 3 of the right TPC. Animation of simulated TPC mode colored by the normal component of displacement (stiff bone: *ρ* = 2400kg/m^3^, *E* = 25GPa, flexible bone: *ρ* = 2000kg/m^3^, *E* = 5GPa).
(MP4)

S4 Video Simulated Mode 3 of the left TPC. Animation of simulated TPC mode colored by the normal component of displacement (stiff bone: *ρ* = 2400kg/m^3^, *E* = 25GPa, flexible bone: *ρ* = 2000kg/m^3^, *E* = 5GPa).
(MP4)

S5 Video Simulated Mode 9 of the right TPC. Animation of simulated TPC mode colored by the normal component of displacement (stiff bone: *ρ* = 2400kg/m^3^, *E* = 25GPa, flexible bone: *ρ* = 2000kg/m^3^, *E* = 5GPa).
(MP4)

S6 Video Simulated Mode 9 of the left TPC. Animation of simulated TPC mode colored by the normal component of displacement (stiff bone: *ρ* = 2400kg/m^3^, *E* = 25GPa, flexible bone: *ρ* = 2000kg/m^3^, *E* = 5GPa).
(MP4)

S7 Video Simulated Mode 11 of the right TPC. Animation of simulated TPC mode colored by the normal component of displacement (stiff bone: *ρ* = 2400kg/m^3^, *E* = 25GPa, flexible bone: *ρ* = 2000kg/m^3^, *E* = 5GPa).
(MP4)

S8 Video Simulated Mode 11 of the left TPC. Animation of simulated TPC mode colored by the normal component of displacement (stiff bone: *ρ* = 2400kg/m^3^, *E* = 25GPa, flexible bone: *ρ* = 2000kg/m^3^, *E* = 5GPa).
(MP4)
