# Supplementary figures and images for: Resonance of the tympanoperiotic complex of fin whales with implications for their low frequency hearing
Source: PLoS One. 2023 Oct 11;18(10):e0288119. doi: 10.1371/journal.pone.0288119 (PMC10566675; doi:10.1371/journal.pone.0288119)

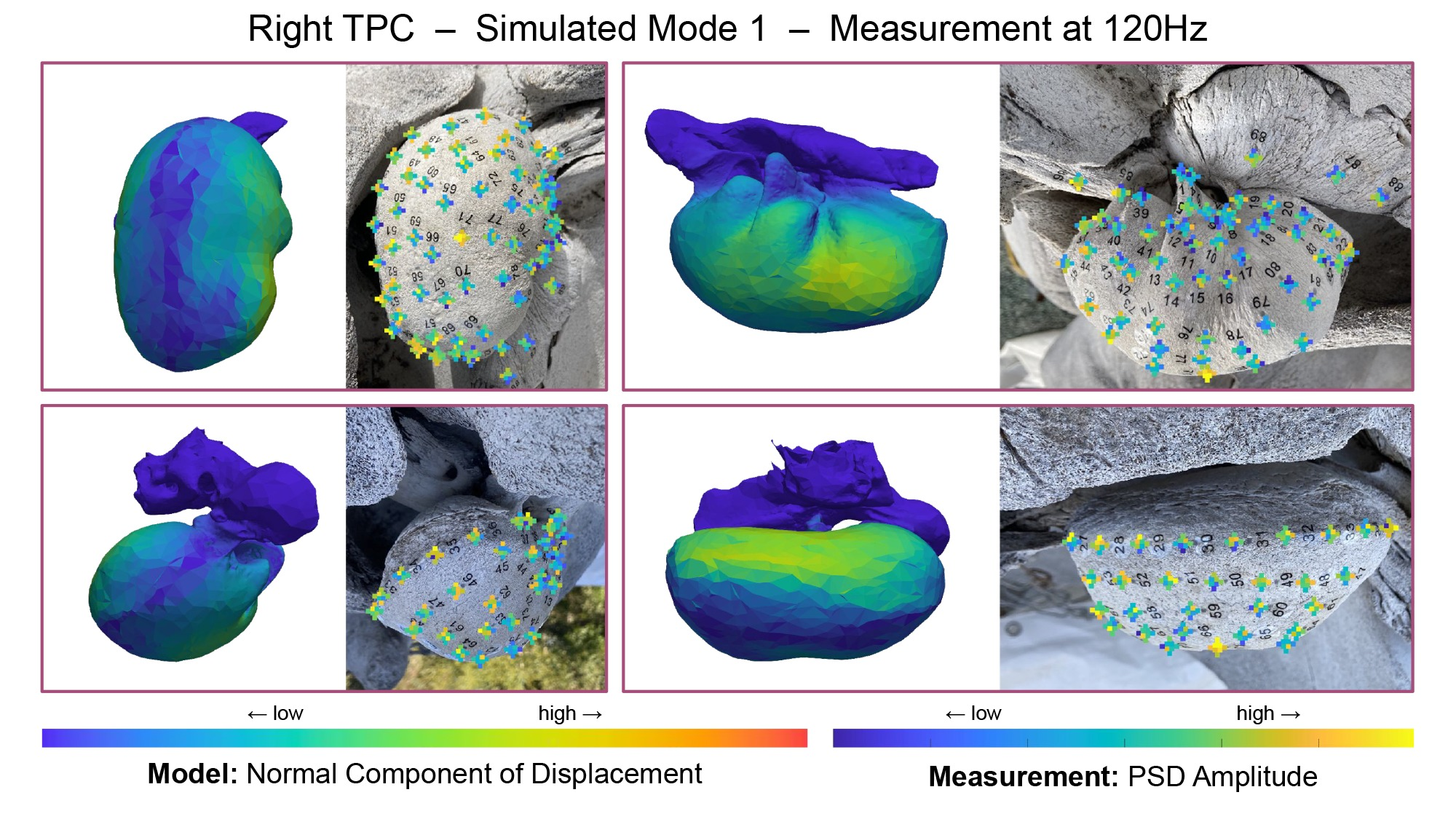

Supplement: S1 Fig — Left on each panel shows the amplitude of the normal component of displacement for the simulated TPC mode (stiff bone: ρ = 2400kg/m3, E = 25GPa, flexible bone: ρ = 2000kg/m3, E = 5GPa). Right on each panel shows an image of the TPC with received amplitudes overlaid as color. (TIF) [file pone.0288119.s001.tif]

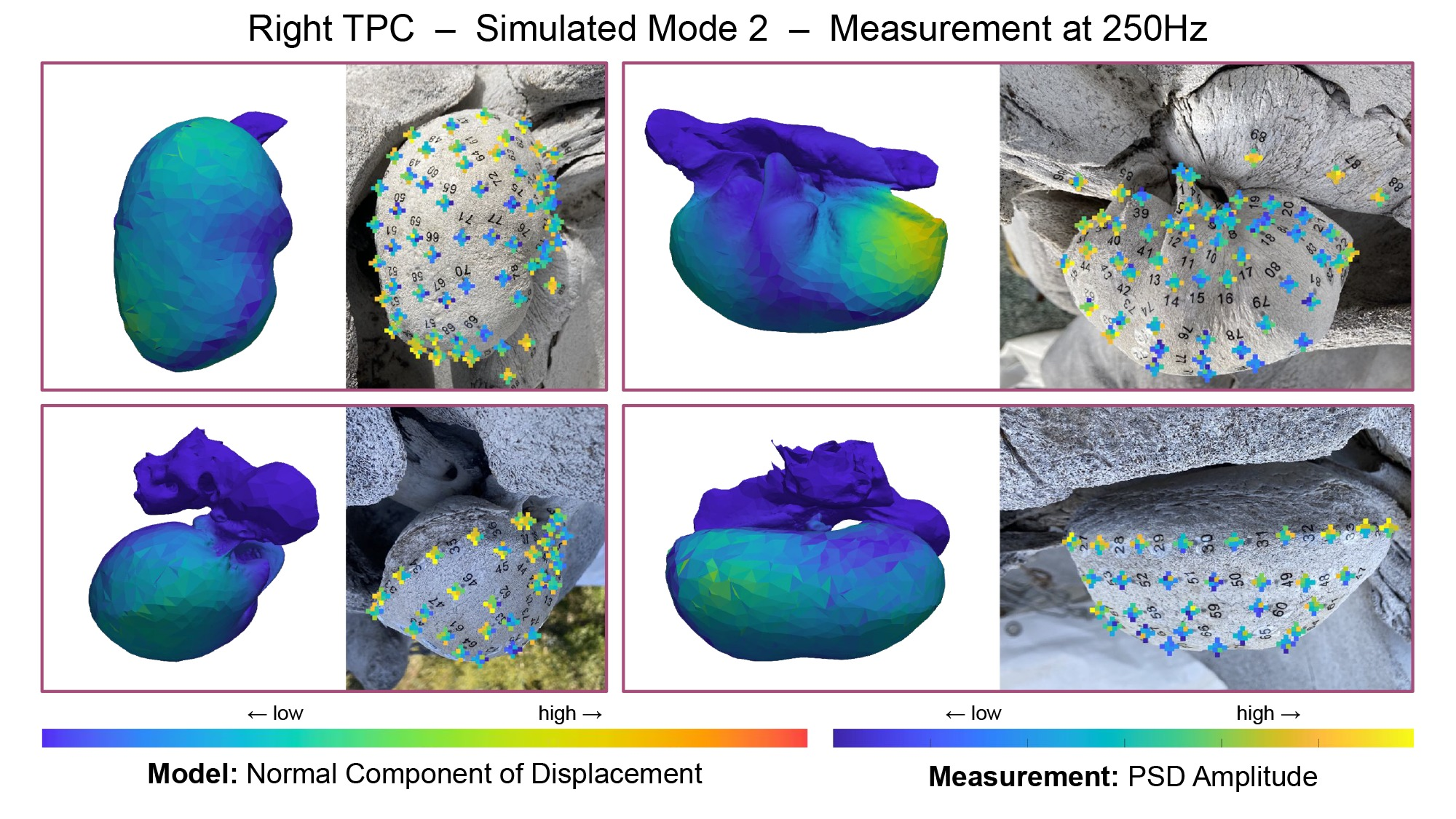

Supplement: S2 Fig — Left on each panel shows the amplitude of the normal component of displacement for the simulated TPC mode (stiff bone: ρ = 2400kg/m3, E = 25GPa, flexible bone: ρ = 2000kg/m3, E = 5GPa). Right on each panel shows an image of the TPC with received amplitudes overlaid as color. (TIF) [file pone.0288119.s002.tif]

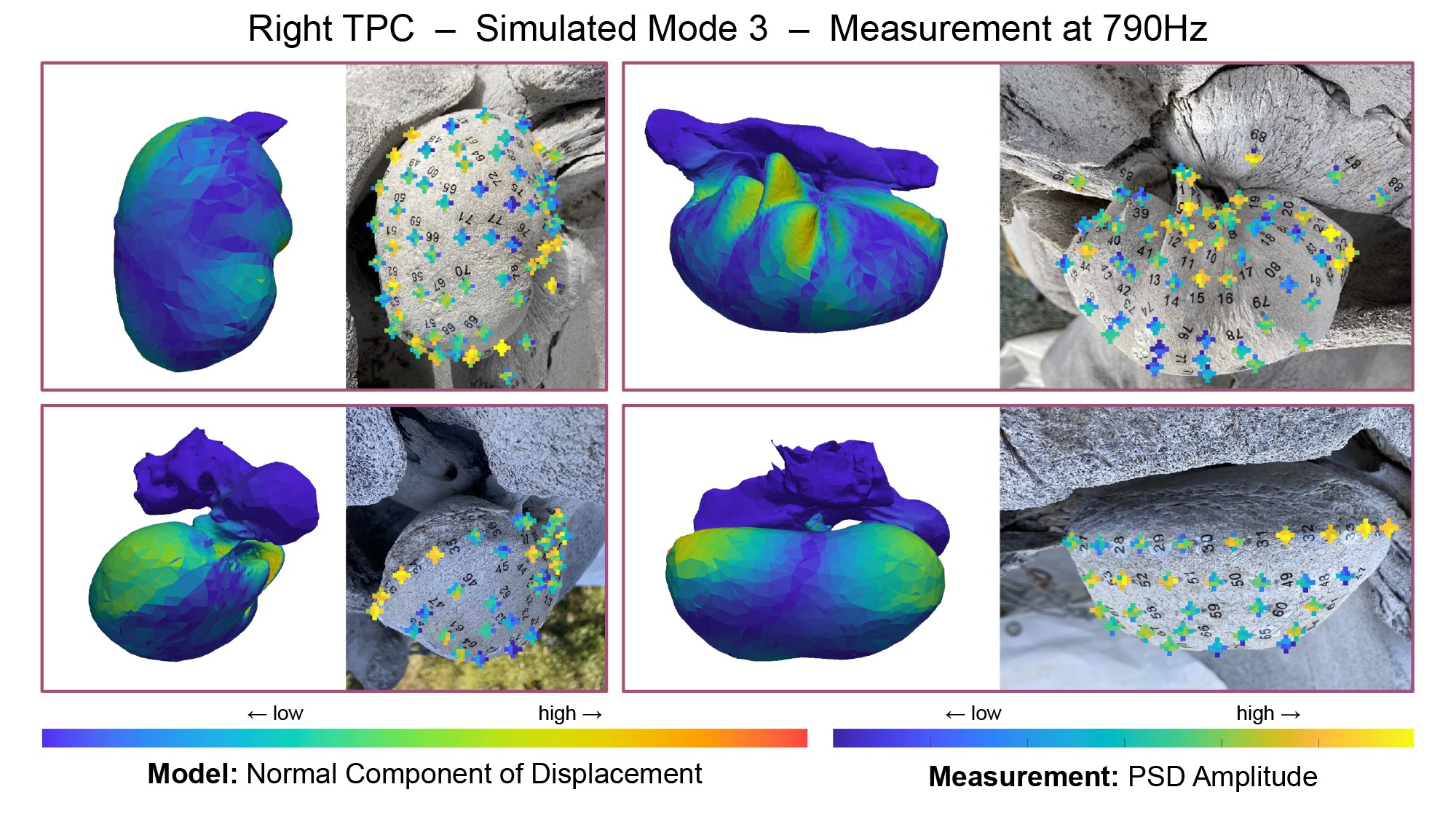

Supplement: S3 Fig — Left on each panel shows the amplitude of the normal component of displacement for the simulated TPC mode (stiff bone: ρ = 2400kg/m3, E = 25GPa, flexible bone: ρ = 2000kg/m3, E = 5GPa). Right on each panel shows an image of the TPC with received amplitudes overlaid as color. (TIF) [file pone.0288119.s003.tif]

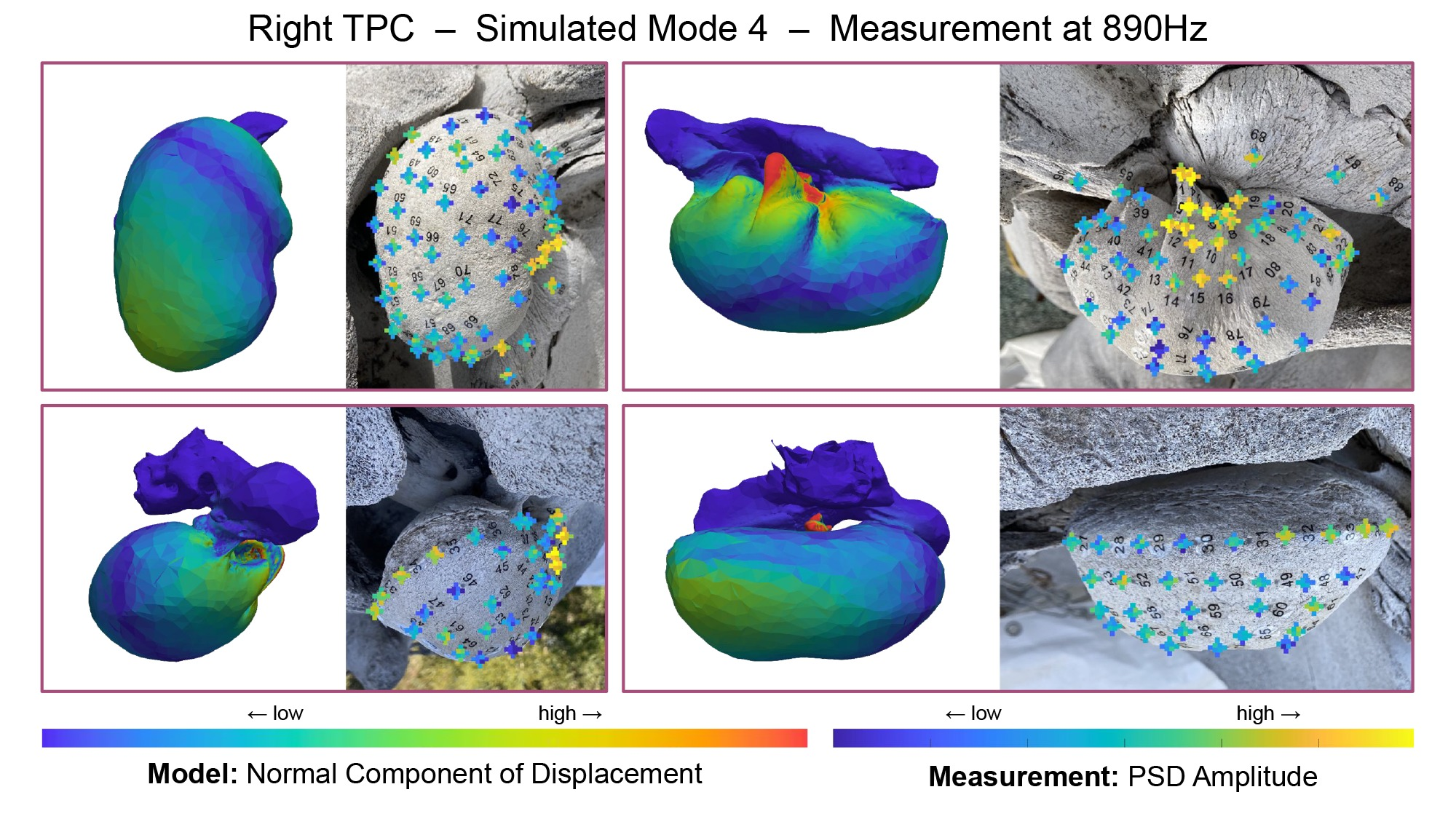

Supplement: S4 Fig — Left on each panel shows the amplitude of the normal component of displacement for the simulated TPC mode (stiff bone: ρ = 2400kg/m3, E = 25GPa, flexible bone: ρ = 2000kg/m3, E = 5GPa). Right on each panel shows an image of the TPC with received amplitudes overlaid as color. (TIF) [file pone.0288119.s004.tif]

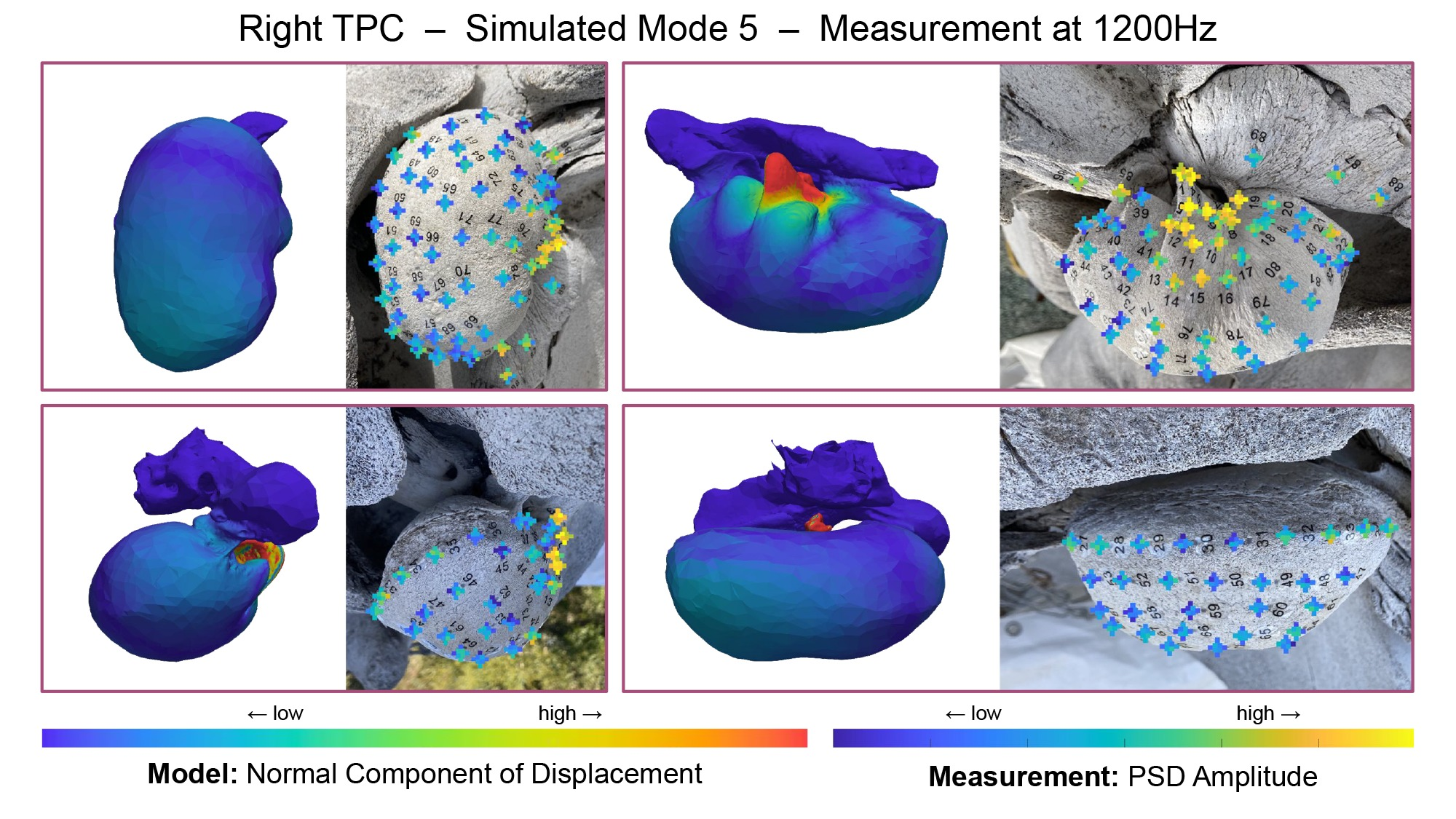

Supplement: S5 Fig — Left on each panel shows the amplitude of the normal component of displacement for the simulated TPC mode (stiff bone: ρ = 2400kg/m3, E = 25GPa, flexible bone: ρ = 2000kg/m3, E = 5GPa). Right on each panel shows an image of the TPC with received amplitudes overlaid as color. (TIF) [file pone.0288119.s005.tif]

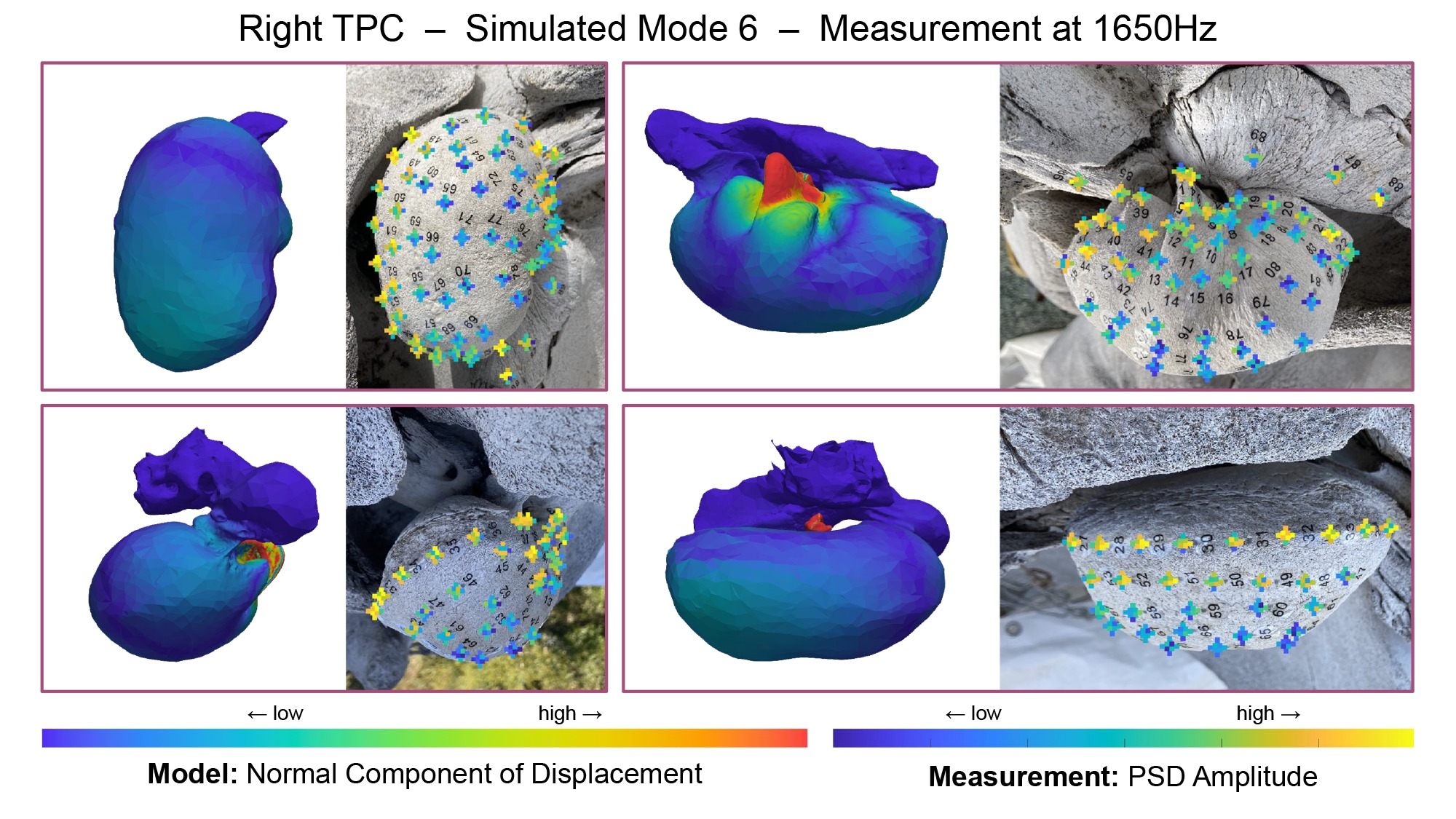

Supplement: S6 Fig — Left on each panel shows the amplitude of the normal component of displacement for the simulated TPC mode (stiff bone: ρ = 2400kg/m3, E = 25GPa, flexible bone: ρ = 2000kg/m3, E = 5GPa). Right on each panel shows an image of the TPC with received amplitudes overlaid as color. (TIF) [file pone.0288119.s006.tif]

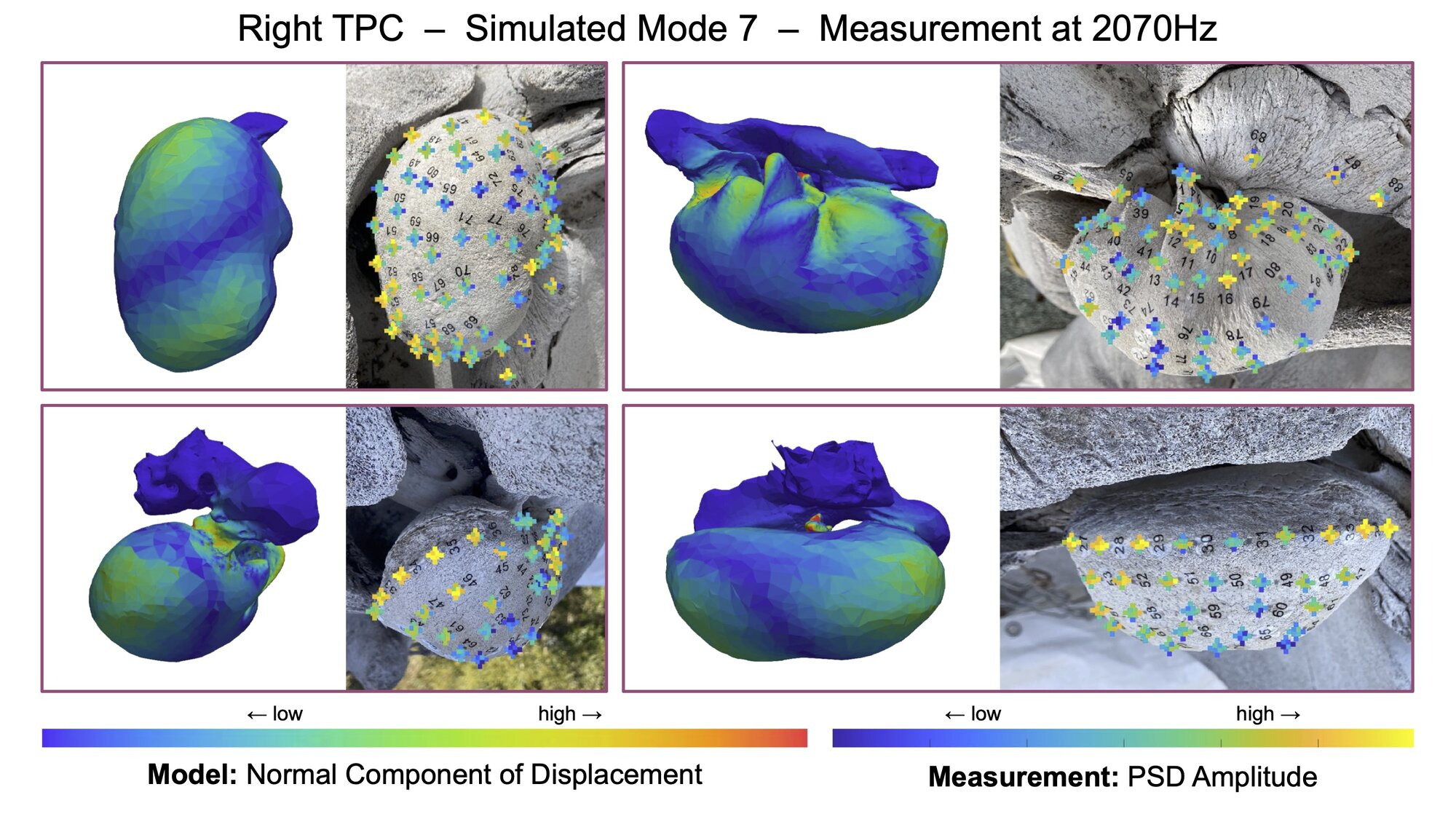

Supplement: S7 Fig — Left on each panel shows the amplitude of the normal component of displacement for the simulated TPC mode (stiff bone: ρ = 2400kg/m3, E = 25GPa, flexible bone: ρ = 2000kg/m3, E = 5GPa). Right on each panel shows an image of the TPC with received amplitudes overlaid as color. (TIF) [file pone.0288119.s007.tif]

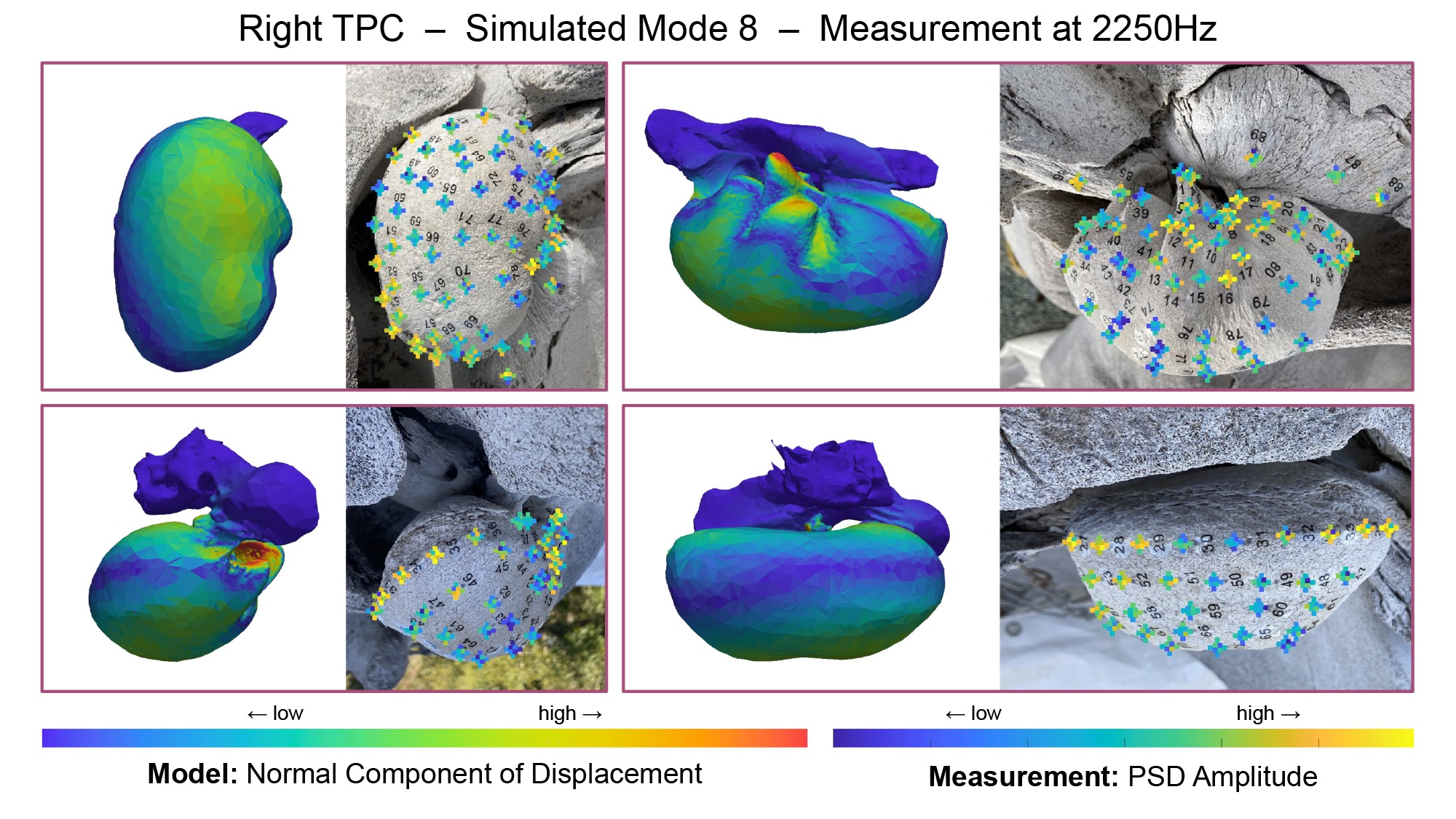

Supplement: S8 Fig — Left on each panel shows the amplitude of the normal component of displacement for the simulated TPC mode (stiff bone: ρ = 2400kg/m3, E = 25GPa, flexible bone: ρ = 2000kg/m3, E = 5GPa). Right on each panel shows an image of the TPC with received amplitudes overlaid as color. (TIF) [file pone.0288119.s008.tif]

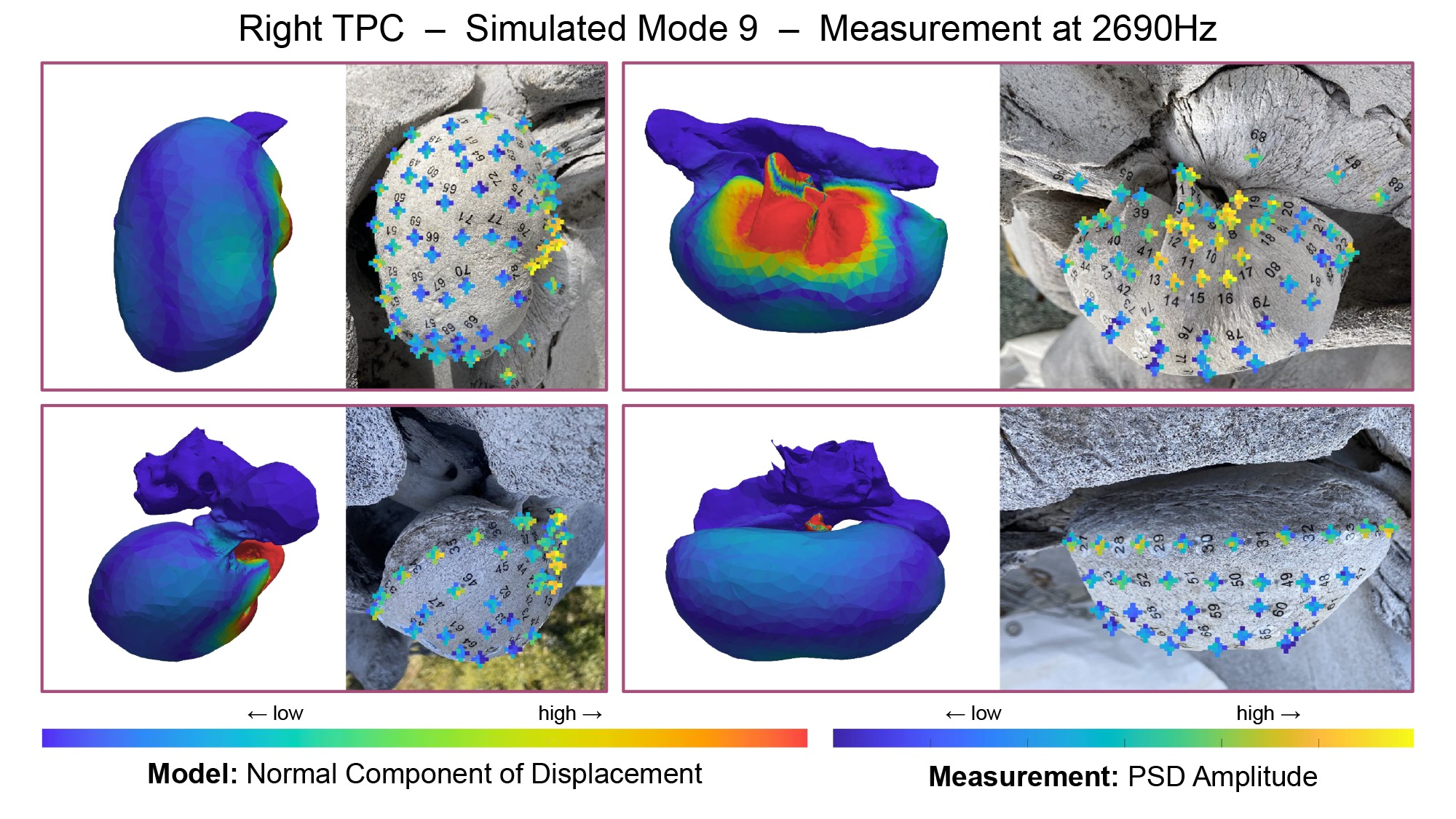

Supplement: S9 Fig — Left on each panel shows the amplitude of the normal component of displacement for the simulated TPC mode (stiff bone: ρ = 2400kg/m3, E = 25GPa, flexible bone: ρ = 2000kg/m3, E = 5GPa). Right on each panel shows an image of the TPC with received amplitudes overlaid as color. (TIF) [file pone.0288119.s009.tif]

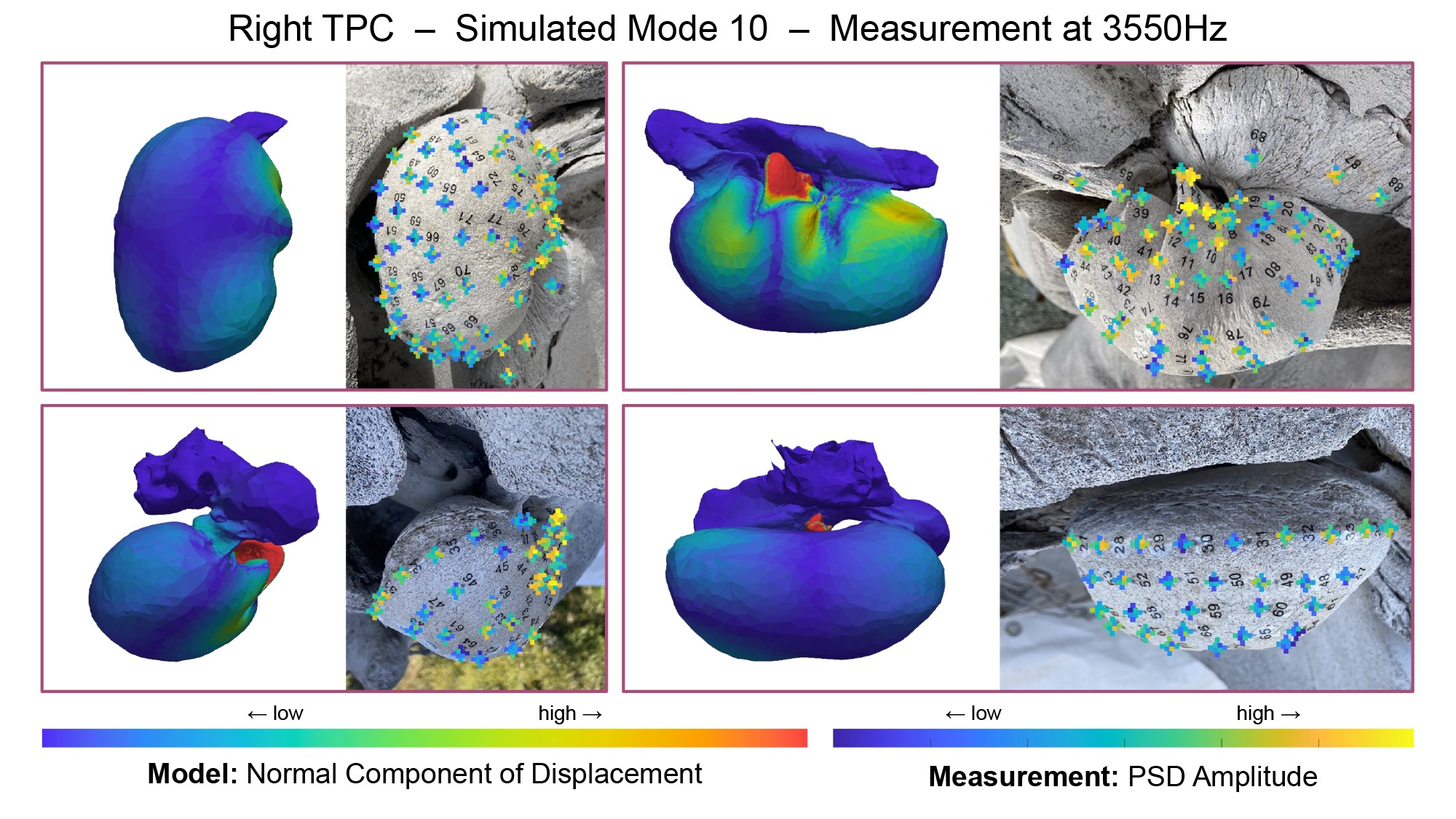

Supplement: S10 Fig — Left on each panel shows the amplitude of the normal component of displacement for the simulated TPC mode (stiff bone: ρ = 2400kg/m3, E = 25GPa, flexible bone: ρ = 2000kg/m3, E = 5GPa). Right on each panel shows an image of the TPC with received amplitudes overlaid as color. (TIF) [file pone.0288119.s010.tif]

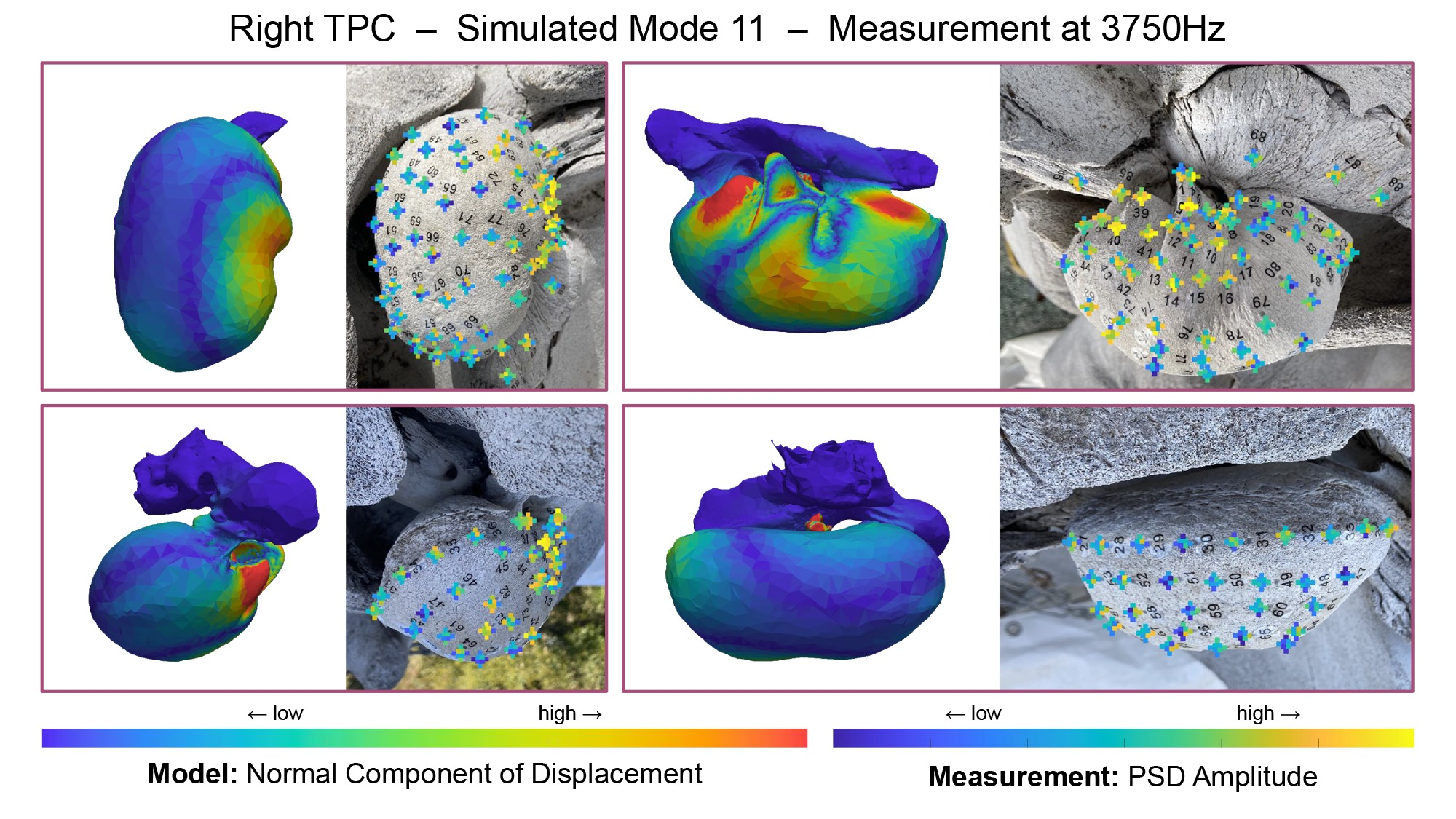

Supplement: S11 Fig — Left on each panel shows the amplitude of the normal component of displacement for the simulated TPC mode (stiff bone: ρ = 2400kg/m3, E = 25GPa, flexible bone: ρ = 2000kg/m3, E = 5GPa). Right on each panel shows an image of the TPC with received amplitudes overlaid as color. (TIF) [file pone.0288119.s011.tif]

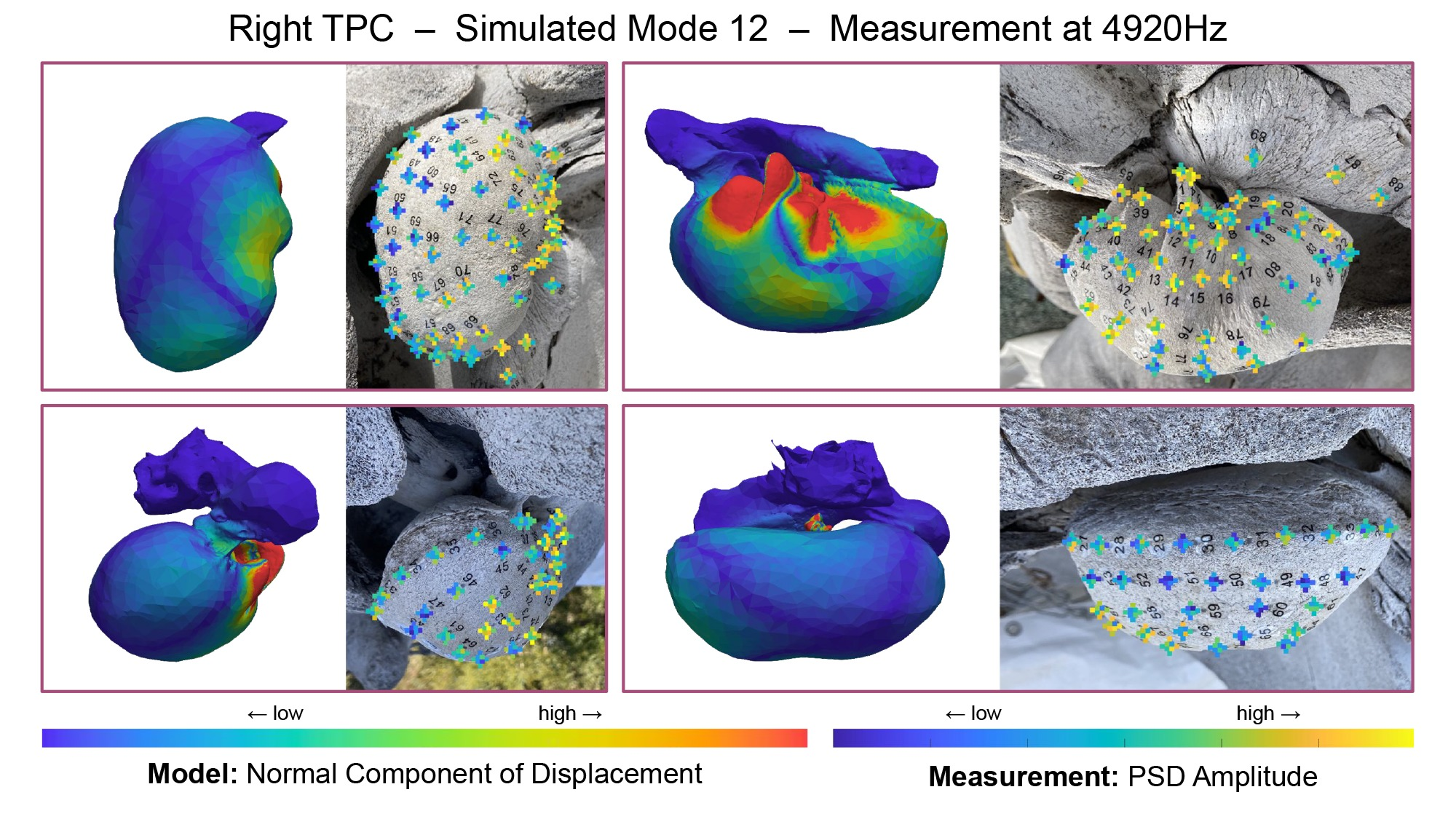

Supplement: S12 Fig — Left on each panel shows the amplitude of the normal component of displacement for the simulated TPC mode (stiff bone: ρ = 2400kg/m3, E = 25GPa, flexible bone: ρ = 2000kg/m3, E = 5GPa). Right on each panel shows an image of the TPC with received amplitudes overlaid as color. (TIF) [file pone.0288119.s012.tif]

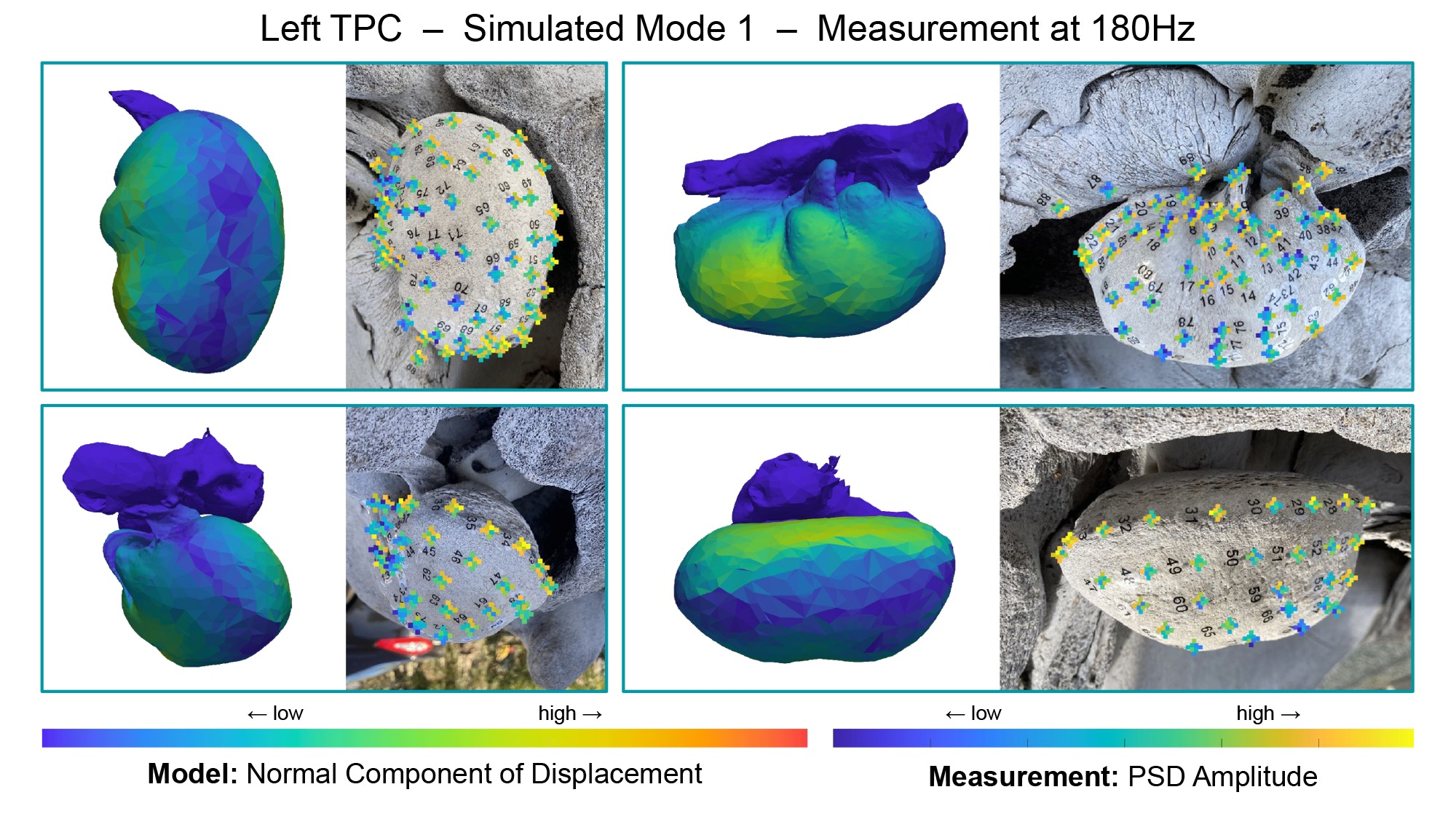

Supplement: S13 Fig — Left on each panel shows the amplitude of the normal component of displacement for the simulated TPC mode (stiff bone: ρ = 2400kg/m3, E = 25GPa, flexible bone: ρ = 2000kg/m3, E = 5GPa). Right on each panel shows an image of the TPC with received amplitudes overlaid as color. (TIF) [file pone.0288119.s013.tif]

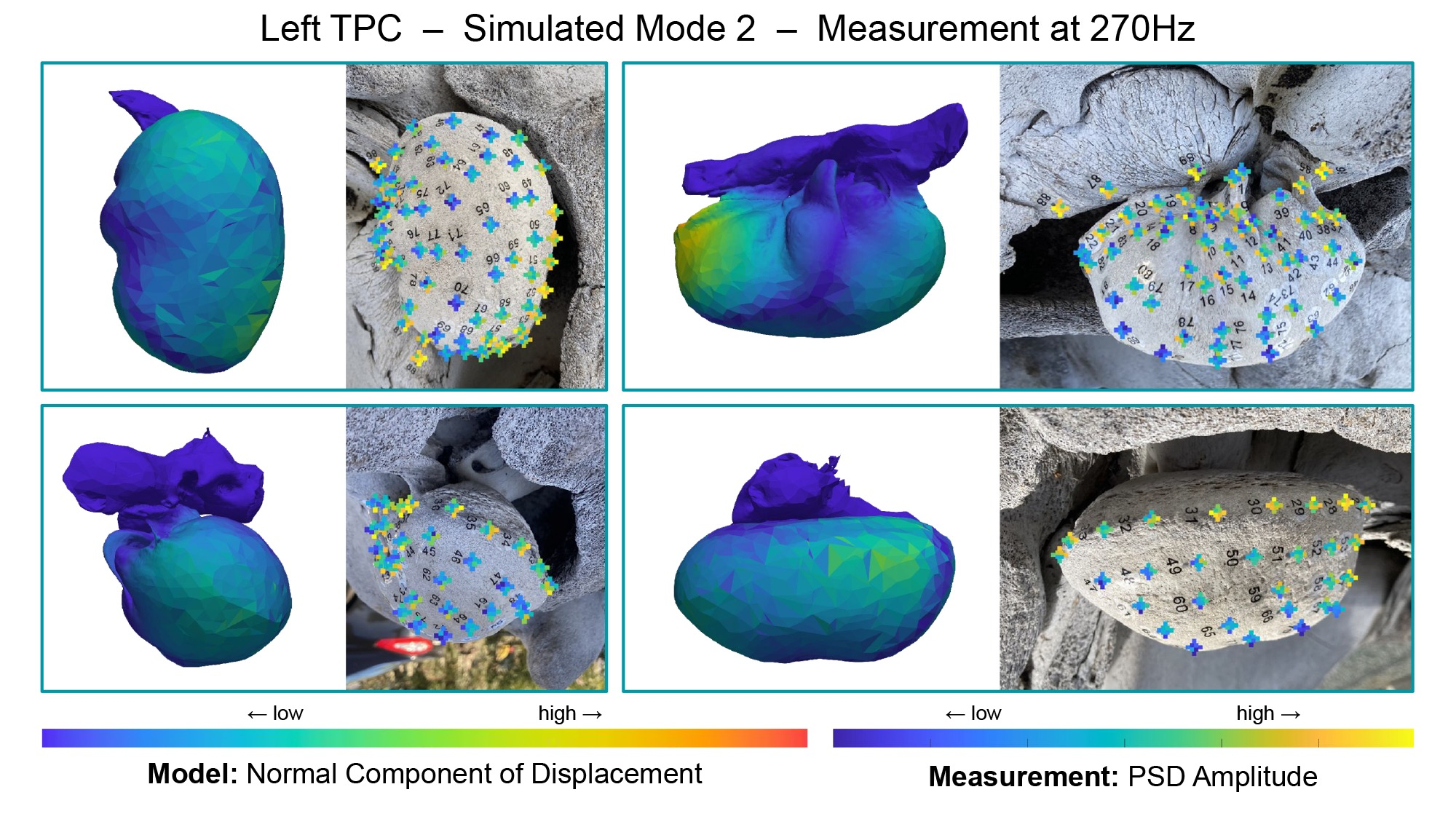

Supplement: S14 Fig — Left on each panel shows the amplitude of the normal component of displacement for the simulated TPC mode (stiff bone: ρ = 2400kg/m3, E = 25GPa, flexible bone: ρ = 2000kg/m3, E = 5GPa). Right on each panel shows an image of the TPC with received amplitudes overlaid as color. (TIF) [file pone.0288119.s014.tif]

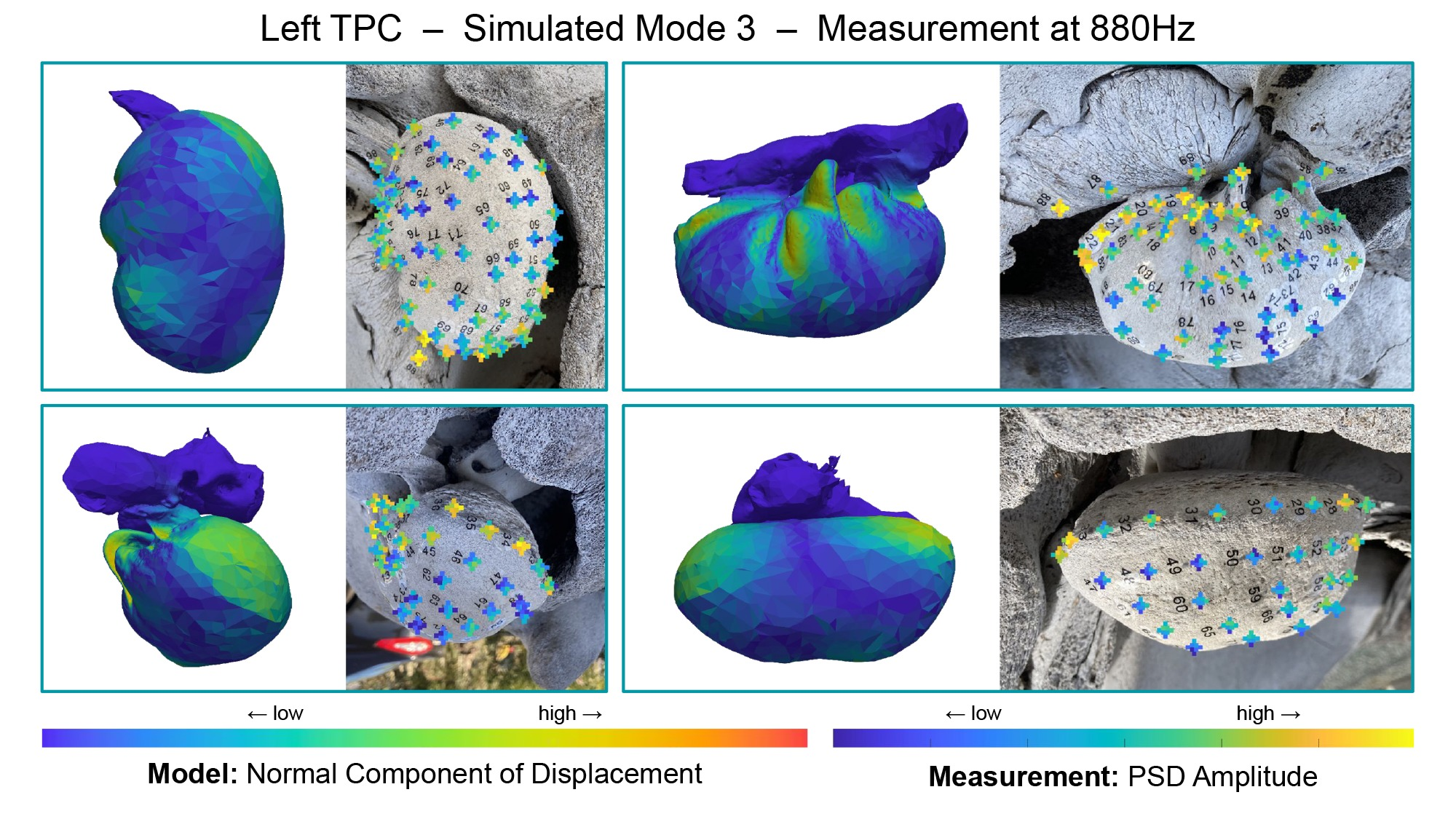

Supplement: S15 Fig — Left on each panel shows the amplitude of the normal component of displacement for the simulated TPC mode (stiff bone: ρ = 2400kg/m3, E = 25GPa, flexible bone: ρ = 2000kg/m3, E = 5GPa). Right on each panel shows an image of the TPC with received amplitudes overlaid as color. (TIF) [file pone.0288119.s015.tif]

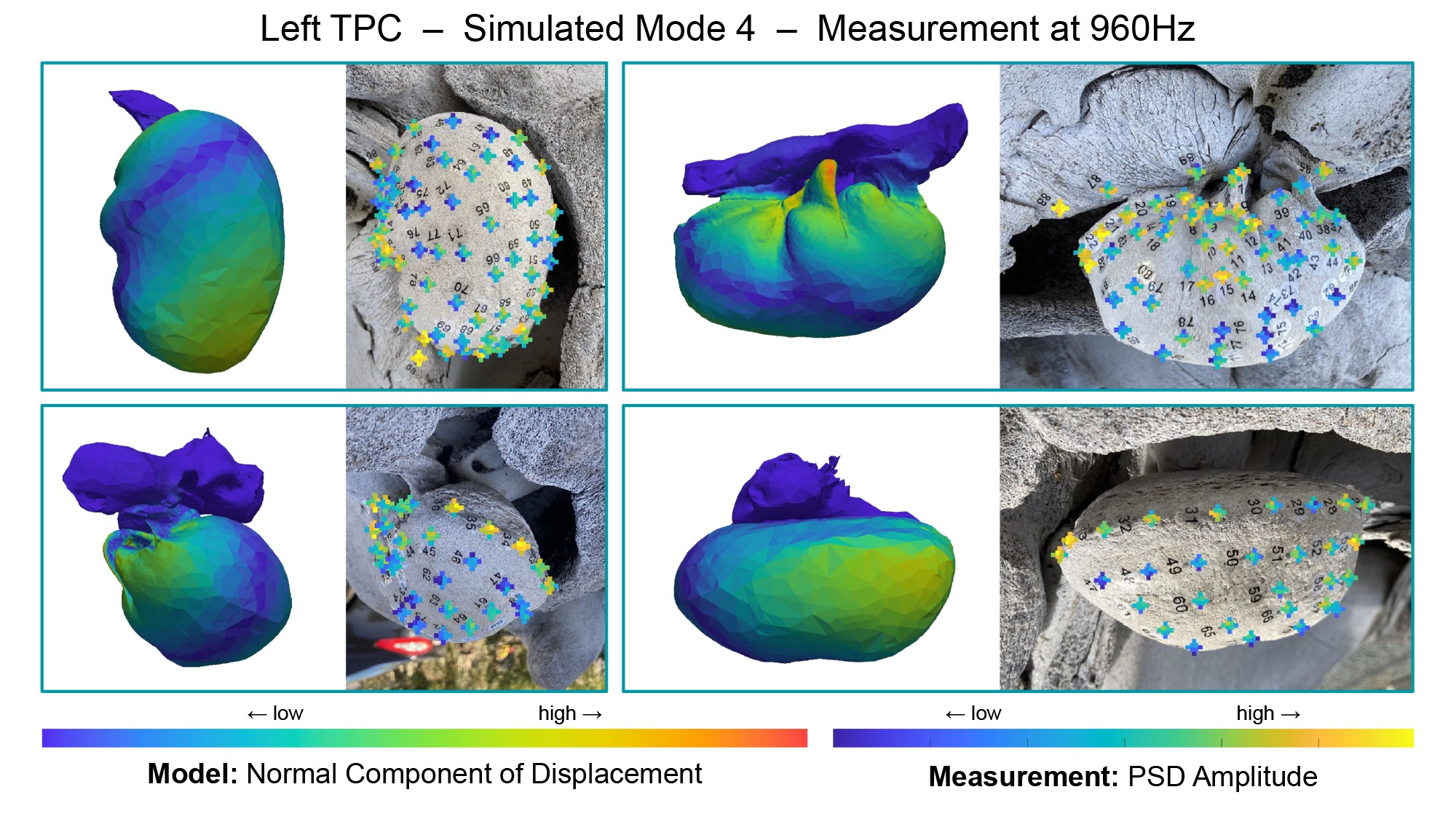

Supplement: S16 Fig — Left on each panel shows the amplitude of the normal component of displacement for the simulated TPC mode (stiff bone: ρ = 2400kg/m3, E = 25GPa, flexible bone: ρ = 2000kg/m3, E = 5GPa). Right on each panel shows an image of the TPC with received amplitudes overlaid as color. (TIF) [file pone.0288119.s016.tif]

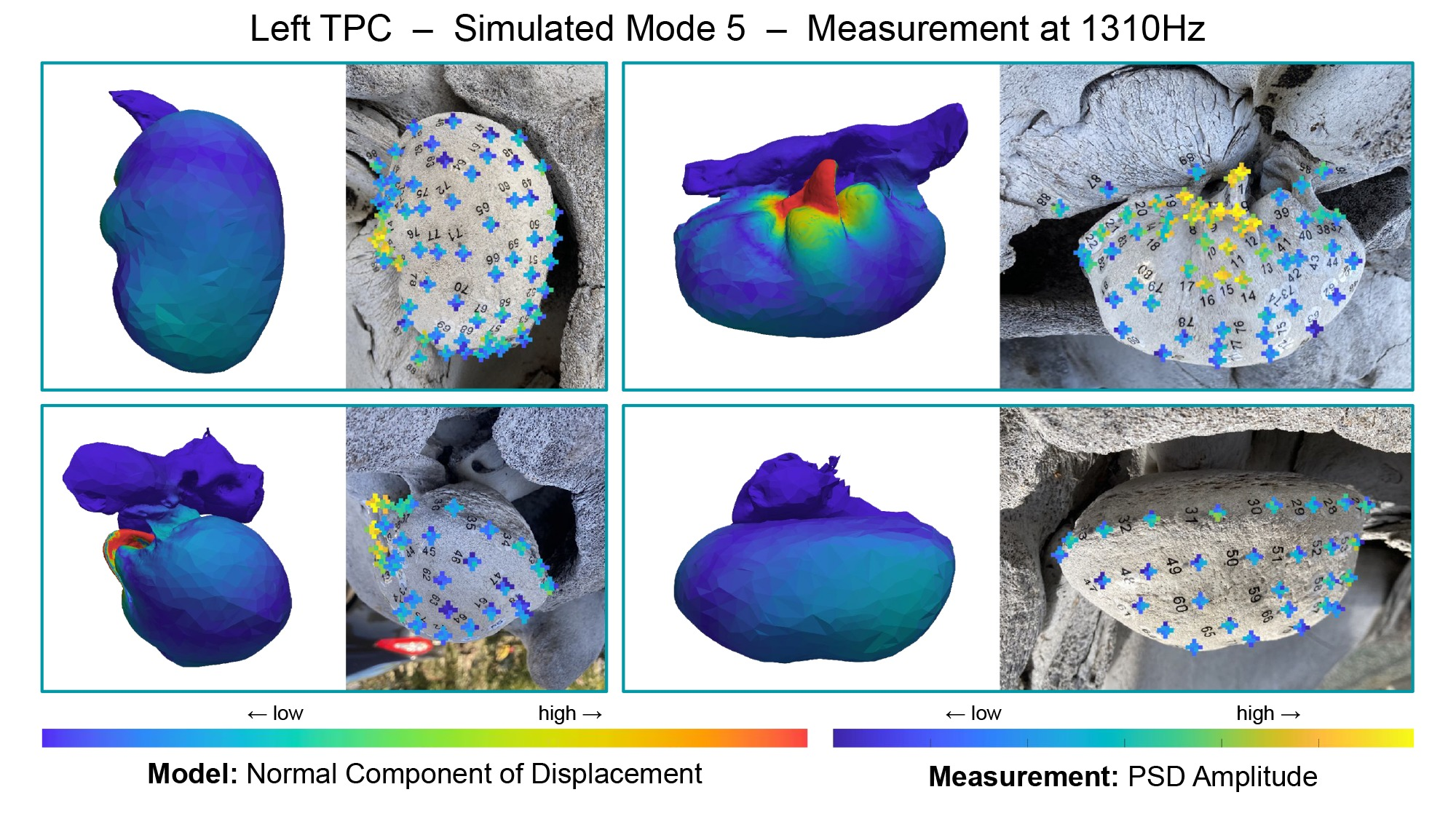

Supplement: S17 Fig — Left on each panel shows the amplitude of the normal component of displacement for the simulated TPC mode (stiff bone: ρ = 2400kg/m3, E = 25GPa, flexible bone: ρ = 2000kg/m3, E = 5GPa). Right on each panel shows an image of the TPC with received amplitudes overlaid as color. (TIF) [file pone.0288119.s017.tif]

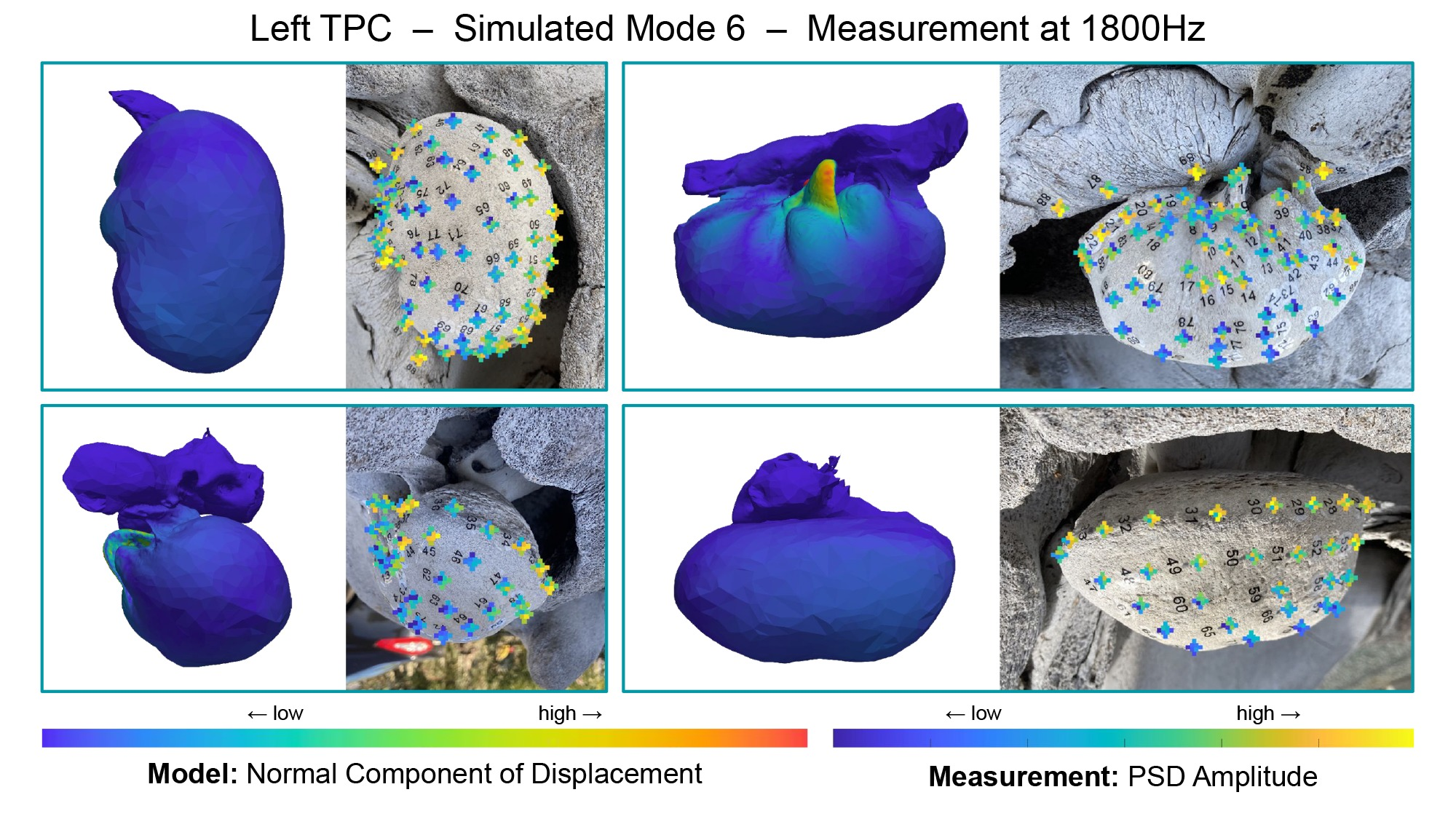

Supplement: S18 Fig — Left on each panel shows the amplitude of the normal component of displacement for the simulated TPC mode (stiff bone: ρ = 2400kg/m3, E = 25GPa, flexible bone: ρ = 2000kg/m3, E = 5GPa). Right on each panel shows an image of the TPC with received amplitudes overlaid as color. (TIF) [file pone.0288119.s018.tif]

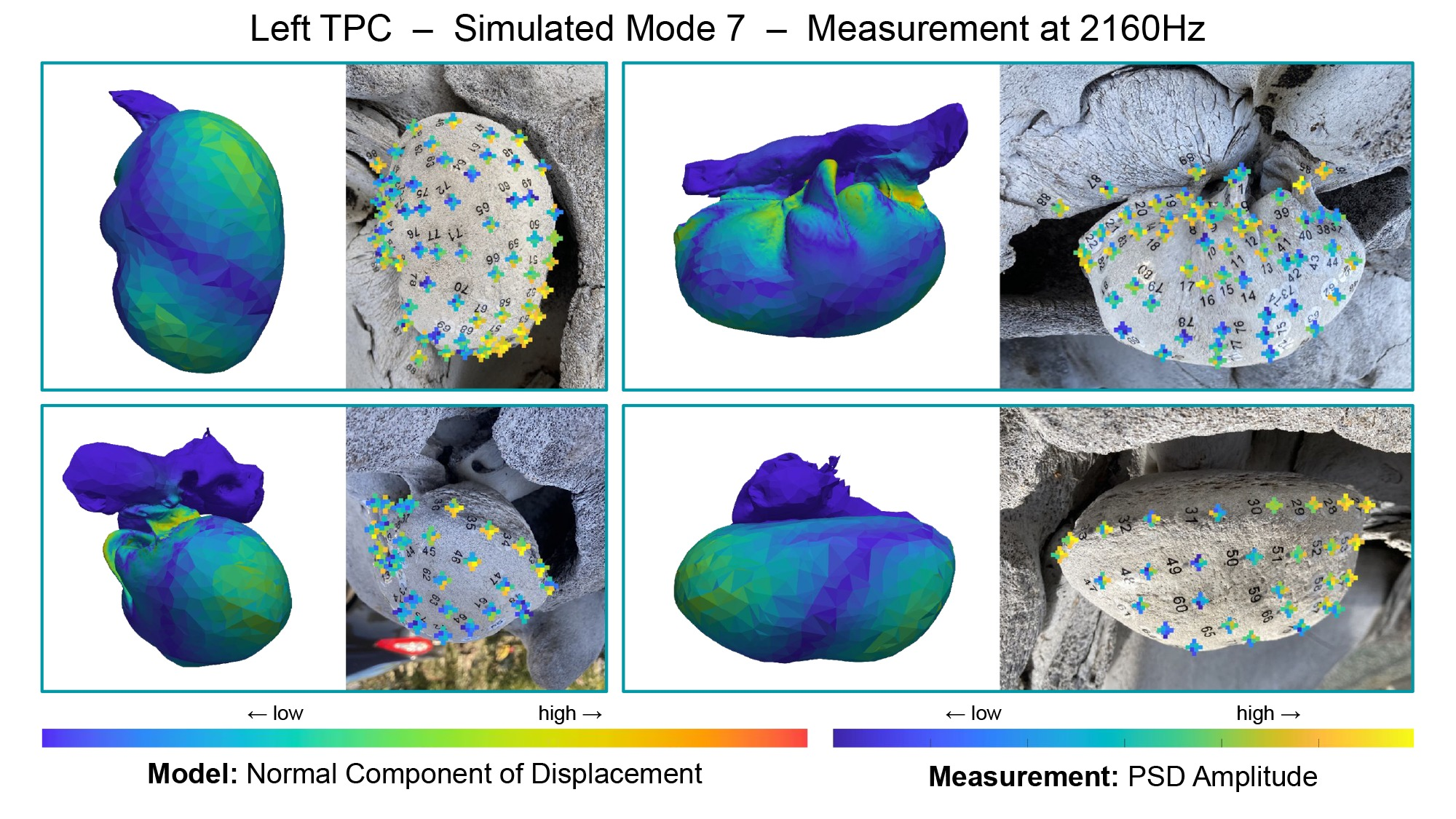

Supplement: S19 Fig — Left on each panel shows the amplitude of the normal component of displacement for the simulated TPC mode (stiff bone: ρ = 2400kg/m3, E = 25GPa, flexible bone: ρ = 2000kg/m3, E = 5GPa). Right on each panel shows an image of the TPC with received amplitudes overlaid as color. (TIF) [file pone.0288119.s019.tif]

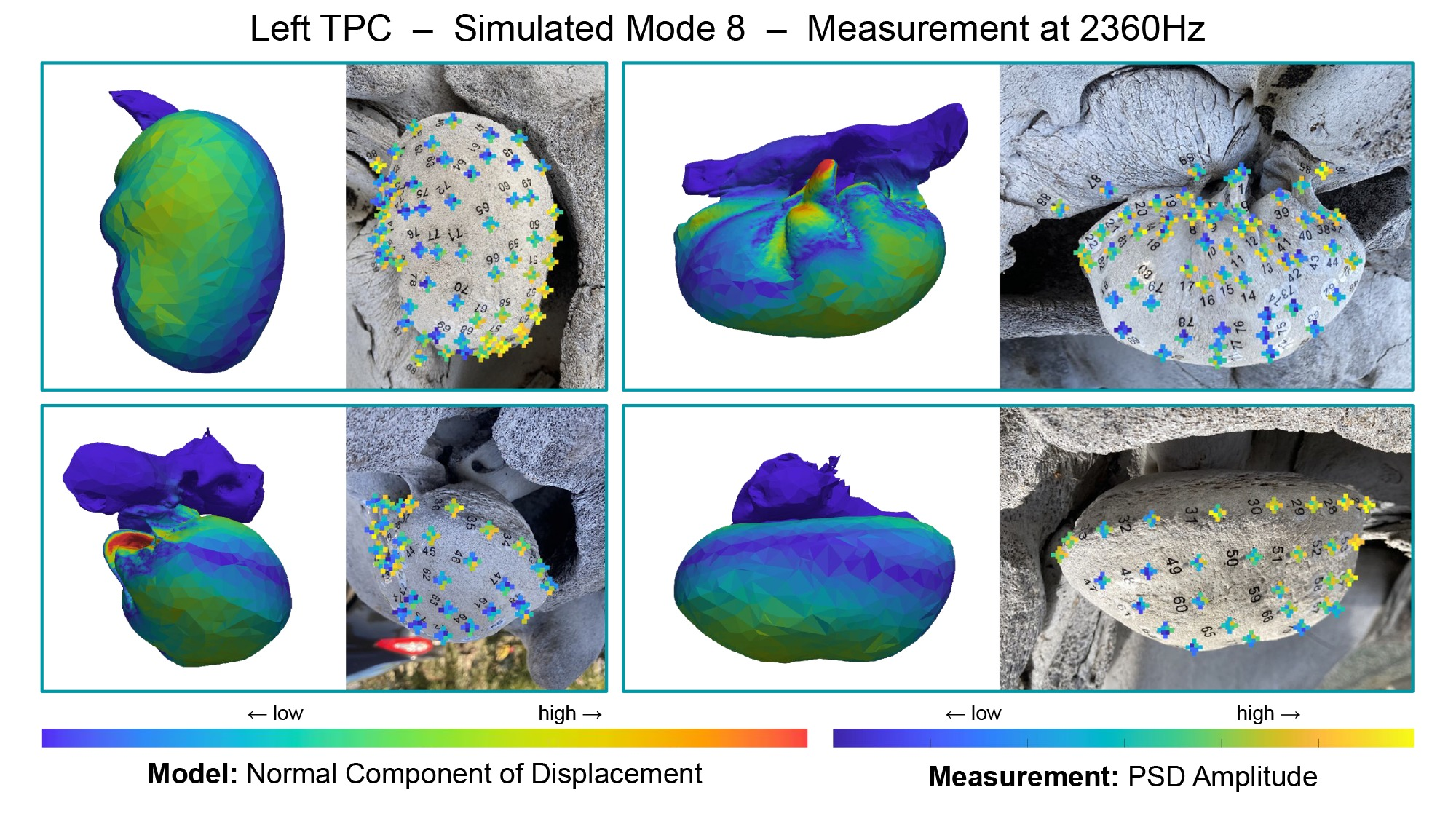

Supplement: S20 Fig — Left on each panel shows the amplitude of the normal component of displacement for the simulated TPC mode (stiff bone: ρ = 2400kg/m3, E = 25GPa, flexible bone: ρ = 2000kg/m3, E = 5GPa). Right on each panel shows an image of the TPC with received amplitudes overlaid as color. (TIF) [file pone.0288119.s020.tif]

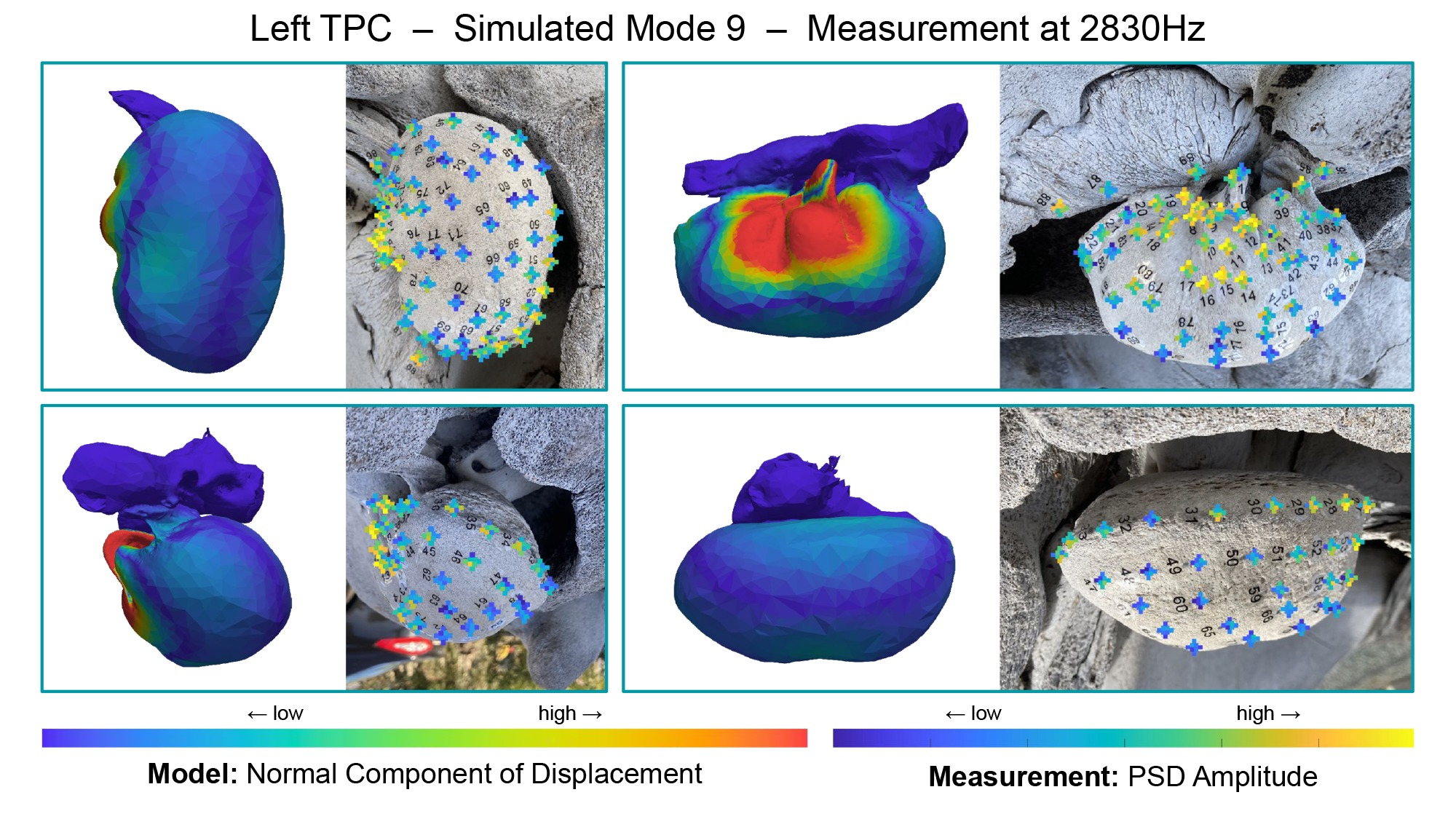

Supplement: S21 Fig — Left on each panel shows the amplitude of the normal component of displacement for the simulated TPC mode (stiff bone: ρ = 2400kg/m3, E = 25GPa, flexible bone: ρ = 2000kg/m3, E = 5GPa). Right on each panel shows an image of the TPC with received amplitudes overlaid as color. (TIF) [file pone.0288119.s021.tif]

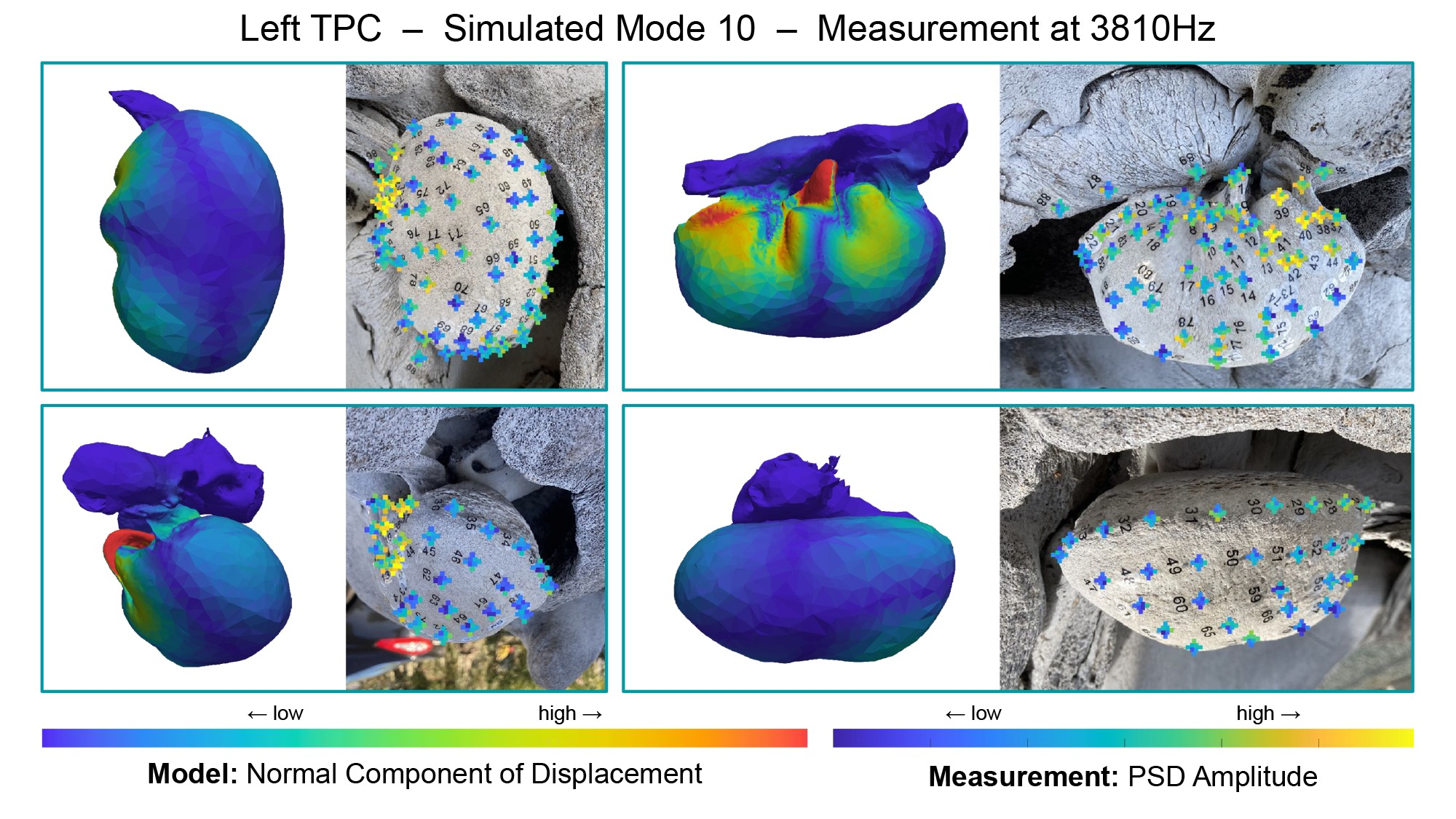

Supplement: S22 Fig — Left on each panel shows the amplitude of the normal component of displacement for the simulated TPC mode (stiff bone: ρ = 2400kg/m3, E = 25GPa, flexible bone: ρ = 2000kg/m3, E = 5GPa). Right on each panel shows an image of the TPC with received amplitudes overlaid as color. (TIF) [file pone.0288119.s022.tif]

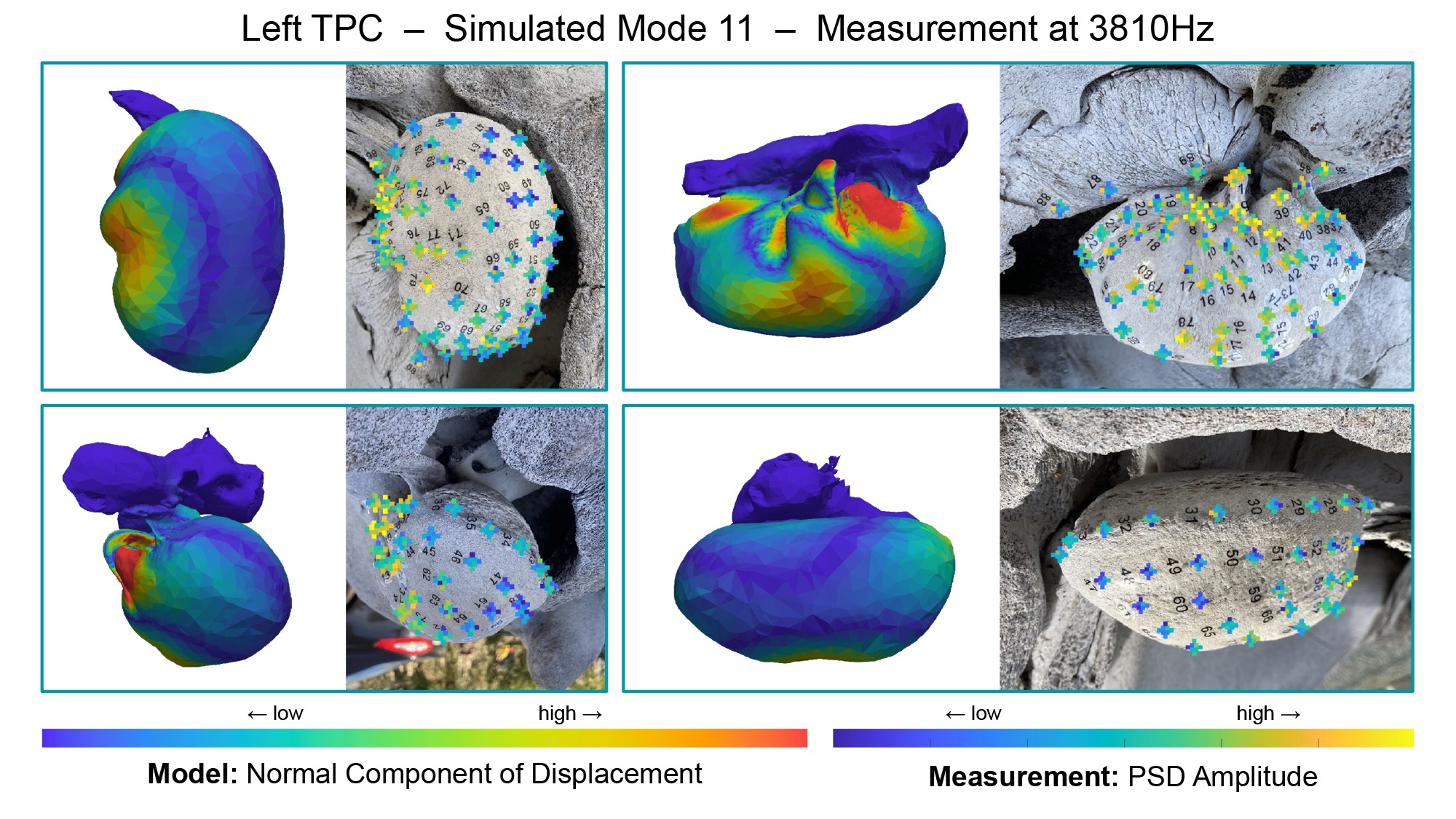

Supplement: S23 Fig — Left on each panel shows the amplitude of the normal component of displacement for the simulated TPC mode (stiff bone: ρ = 2400kg/m3, E = 25GPa, flexible bone: ρ = 2000kg/m3, E = 5GPa). Right on each panel shows an image of the TPC with received amplitudes overlaid as color. (TIF) [file pone.0288119.s023.tif]

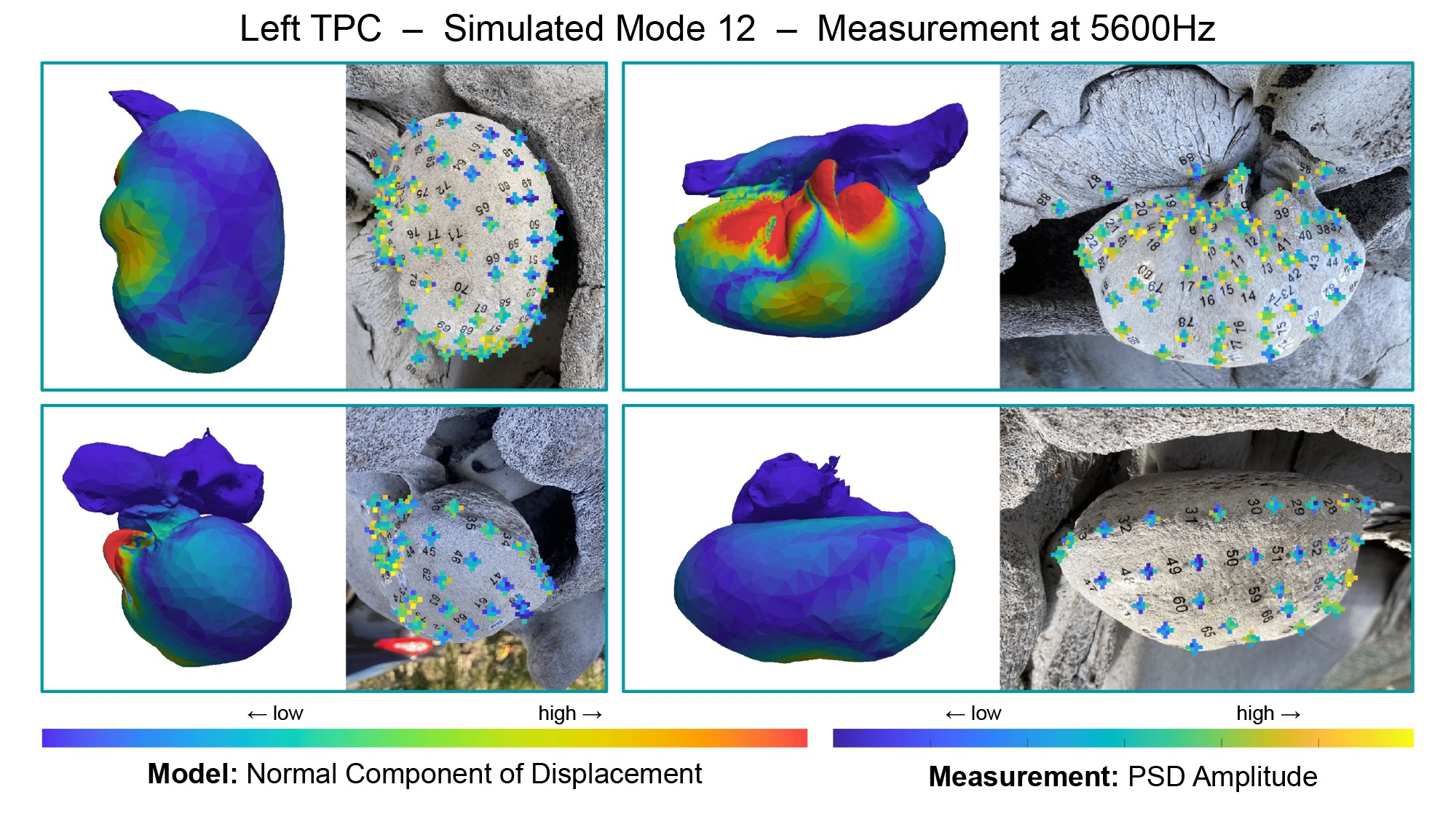

Supplement: S24 Fig — Left on each panel shows the amplitude of the normal component of displacement for the simulated TPC mode (stiff bone: ρ = 2400kg/m3, E = 25GPa, flexible bone: ρ = 2000kg/m3, E = 5GPa). Right on each panel shows an image of the TPC with received amplitudes overlaid as color. (TIF) [file pone.0288119.s024.tif]
